# ppGalNAc-T4-catalyzed O-Glycosylation of TGF-β type Ⅱ receptor regulates breast cancer cells metastasis potential

**DOI:** 10.1074/jbc.RA120.016345

**Published:** 2020-12-03

**Authors:** Qiong Wu, Cheng Zhang, Keren Zhang, Qiushi Chen, Sijin Wu, Huang Huang, Tianmiao Huang, Nana Zhang, Xue Wang, Wenli Li, Yubo Liu, Jianing Zhang

**Affiliations:** 1 School of Life Science & Pharmacy, Dalian University of Technology, Panjin, China; 2 Clinical Laboratory of BGI Health, BGI-Shenzhen, Shenzhen, China; 3 Division of Medicinal Chemistry and Pharmacognosy, College of Pharmacy, The Ohio State University, Columbus, Ohio, USA; 4 Laboratory of Molecular Modeling and Design, State Key Laboratory of Molecular Reaction Dynamics, Dalian Institute of Chemical Physics, Chinese Academy of Sciences, Dalian, China

**Keywords:** ppGalNAc-T4, O-GalNAc glycosylation, epithelial–mesenchymal transition (EMT), TGF-β type Ⅰ and Ⅱ receptors, breast cancer, BL, basal-like, CL, claudin-low, co-IP, coimmunoprecipitation, CRC, colorectal cancer, DMEM, Dulbecco’s modified Eagle’s medium, EMT, epithelial–mesenchymal transition, FBS, fetal bovine serum, GEO, Gene Expression Omnibus, GEPIA, Gene Expression Profiling Interactive Analysis, IP, immunoprecipitation, MD, molecular dynamics, PDAC, pancreatic ductal adenocarcinoma, PDB, Protein Data Bank, RFS, recurrence-free survival, shRNA, short hairpin RNA, TGF-β, transforming growth factor beta, VVL, *Vicia villosa* lectin, WT, wild-type

## Abstract

GalNAc-type O-glycosylation, initially catalyzed by polypeptide N-acetylgalactosaminyltransferases (ppGalNAc-Ts), is one of the most abundant and complex posttranslational modifications of proteins. Emerging evidence has proven that aberrant ppGalNAc-Ts are involved in malignant tumor transformation. However, the exact molecular functions of ppGalNAc-Ts are still unclear. Here, the role of one isoform, ppGalNAc-T4, in breast cancer cell lines was investigated. The expression of ppGalNAc-T4 was found to be negatively associated with migration of breast cancer cells. Loss-of-function studies revealed that ppGalNAc-T4 attenuated the migration and invasion of breast cancer cells by inhibiting the epithelial–mesenchymal transition (EMT) process. Correspondingly, transforming growth factor beta (TGF-β) signaling, which is the upstream pathway of EMT, was impaired by ppGalNAc-T4 expression. ppGalNAc-T4 knockout decreased O-GalNAc modification of TGF-β type Ⅰ and Ⅱ receptor (TβR Ⅰ and Ⅱ) and led to the elevation of TGF-β receptor dimerization and activity. Importantly, a peptide from TβR Ⅱ was identified as a naked peptide substrate of ppGalNAc-T4 with a higher affinity than ppGalNAc-T2. Further, Ser31, corresponding to the extracellular domain of TβR Ⅱ, was identified as the O-GalNAcylation site upon *in vitro* glycosylation by ppGalNAc-T4. The O-GalNAc-deficient S31 A mutation enhanced TGF-β signaling activity and EMT in breast cancer cells. Together, these results identified a novel mechanism of ppGalNAc-T4-catalyzed TGF-β receptors O-GalNAcylation that suppresses breast cancer cell migration and invasion *via* the EMT process. Targeting ppGalNAc-T4 may be a potential therapeutic strategy for breast cancer treatment.

Breast cancer, one of the most commonly diagnosed malignancies, accounts for 24.2% of female cancers and is the leading cause of cancer-related death among women worldwide (1). Despite advances in early diagnosis and treatment, a high rate of metastasis remains the underlying cause of death in the majority of patients (2). Therefore, elucidation of the underlying molecular mechanisms of breast cancer metastasis and development of efficient prognostic and therapeutic biomarkers are urgently needed.

The epithelial-to-mesenchymal transition (EMT) process is necessary for tumor progression toward metastatic disease, during which cells lose polarity and cell–cell junctions and gain mesenchymal properties. EMT promotes cancer cell motility and dissemination (3, 4). The transforming growth factor-β (TGF-β) signaling pathway evidently increases tumor malignancy in advanced cancer by inducing EMT in response to TGF-β (5). Signal transduction occurs upon ligand binding with heteromeric complexes of TGF-β type Ⅰ and R Ⅱ receptor (TβR Ⅰ and TβR Ⅱ) to induce Smad2/3 phosphorylation, which activates Smad4 accumulation in the nucleus to regulate downstream transcription factors (6). These targeted genes, especially SNAI1 and TCF8, induce EMT as E-cadherin repressors (7). Recently, emerging evidence has shown that aberrant glycosylation is involved in cancer EMT and metastasis (8, 9), but the biological roles of these modifications remain mostly unknown.

Glycosylation, as a kind of protein posttranslational modification, is a stepwise process of covalent attachment of oligosaccharide chains to polypeptides or lipids that is strictly regulated by the cooperation of glycosyltransferases and glycosidases. Abnormal glycosylation catalyzed by altered glycosyltransferases is a hallmark of cancer. Aberrant sugar chain structures and glycoproteins play vital roles in cancer pathological events, including transformation and metastasis (10, 11). GalNAc-type O-glycosylation is emerging as one of the most abundant and diverse modifications affecting the majority of membrane and secreted proteins and is involved in many biological activities in cancers (12, 13, 14, 15). In animals, O-GalNAcylation is initiated by a family of up to 20 homologous genes encoding polypeptide N-acetylgalactosaminyltransferases (ppGalNAc-Ts), each displaying selective tissue and substrate protein specificity (16). Aberrant O-GalNAcylation and ppGalNAc-Ts represent potential markers associated with poor prognosis and tumor metastasis. Tn antigen (GalNAcα1-O-Ser/Thr) and T antigen (Galβ1-3GalNAcα1-O-Ser/Thr), uncovered at high levels in most primary and metastatic carcinomas, are involved in invasion (17, 18). As examples, in pancreatic ductal adenocarcinoma (PDAC), truncated O-linked GalNAc glycosylation frequently showed alteration resulting in profound cellular changes (19), and loss of ppGalNAc-T3 was reported to be associated with increased aggressiveness in PDAC (20). Overexpression of ppGalNAc-T6 dramatically inhibited cellular colony formation, migration, and invasion and promoted the apoptosis of colorectal cancer cells as a tumor suppressor (21). Increased ppGalNAc-T2 expression is associated with an unfavorable prognosis and a higher tumor grade in human gliomas and facilitates the malignant characteristics of glioma (22). In oral squamous cell carcinoma, ppGalNAc-T2 enhances the invasive potential by modifying the O-glycosylation of EGFR, which could be a promising therapeutic approach (23). Some specific ppGalNAc-T isoforms have been reported to be relevant to EMT and/or TGF-beta signaling. ppGalNAc-T3 and ppGalNAc-T6 have been suggested as enzymes involved in TGF-β-induced onfFN and EMT processes (24, 25, 26, 27), and ppGalNAc-T6 was determined to be an early marker of EMT (28). ppGalNAc-T14 plays a critical role in the invasion and migration of breast cancer cells by regulating the activity of MMP-2 and the expression of some EMT genes (29). xGalNAc-T6 and xGalNAc-T16 from the African clawed frog (*Xenopus laevis*), which show high homology to human ppGalNAc-T6 and ppGalNAc-T16, had contrasting roles in TGF-β/BMP signaling in embryogenesis (30). Recently, accumulating reports have demonstrated that ppGalNAc-T4 participates in numerous cellular processes, including tumorigenesis (31, 32, 33). In hepatocellular carcinoma, ppGalNAc-T4 was reported attenuating cellular migration, invasion, and stemness and inducing anoikis of HCC cells *in vitro* (34). To date, although there is evidence showing that ppGalNAc-T4 can catalyze naked EA2 peptide from rat submandibular mucin (35), other reports reveal that this enzyme prefers to recognize GalNAc-glycosylated substrates (36), where prior glycosylation has been catalyzed by other GalNAc-transferase isoforms (*e.g*., ppGalNAc-T1, -T2, and -T3) (37, 38). However, the exact molecular function and potential substrates of ppGalNAc-T4 remain mostly unknown in human breast cancer.

Herein, the role of ppGalNAc-T4 in breast cancer was investigated. Loss-of-function studies demonstrated that ppGalNAc-T4 inhibited the migration and invasion of breast cancer cells by suppressing TGF-β/Smad signaling-induced EMT. Importantly, for the first time, we demonstrated that ppGalNAc-T4 modulated TGF-β signaling by directly catalyzing O-GalNAcylation of TGF-β type Ⅱ receptor at Ser31 and then attenuated dimerization of TGF-β receptors, resulting in the inhibition of TGF-β signaling and EMT in breast cancer cells. Together, these results unravel a novel mechanism of ppGalNAc-T4 suppressing breast cancer cell migration and invasion. Targeting ppGalNAc-T4 may be a potential therapeutic strategy for breast cancer treatment.

## Results

### The expression of ppGalNAc-T4 negatively correlates with the metastatic capacity of human breast cancer

**Figure 1:**
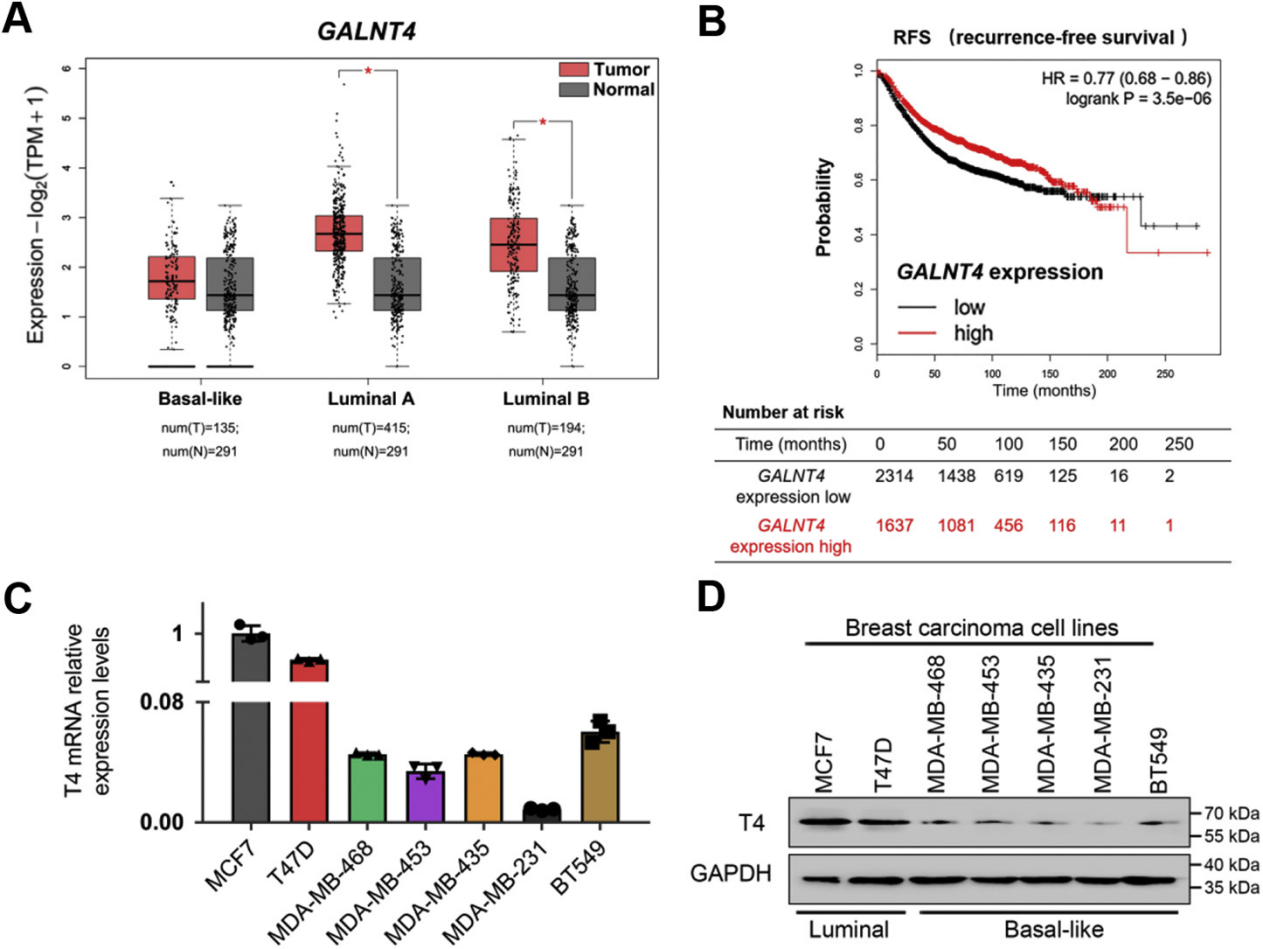
**The expression of ppGalNAc-T4 negatively correlates with human breast cancer metastasis capacity.***A*, box–whisker plot represented expression of *GALNT4* in breast carcinoma based on subtypes compared with normal controls determined by GEPIA database. *Red* represents tumor tissue, *black* represents normal tissue. *B*, RFS curves were plotted for breast carcinoma patients. Data was analyzed using Kaplan–Meier Plotter. Patients with *GALNT4* expression above the median are indicated in red line, and patients with *GALNT4* expressions below the median in *black line*. *P* values by log-rank test; ∗*p* < 0.05. *C–D*, qPCR and western blot analysis of endogenous ppGalNAc-T4 expression in different human breast cancer cell lines. MCF7 and T47D belong to low-metastatic Lum subtype breast cancer; MDA-MB-468, MDA-MB-453, MDA-MB-435, MDA-MB-231, and BT549 belong to highly invasive basal-like subtype. The data were obtained from three independent experiments, presented as mean ± SD. *P* values by paired *t*-test; ∗*p* < 0.05; GAPDH was used as the internal control.

Given the distinct substrate recognition specificity, the function and mechanism of ppGalNAc-T4 in cancer progression might be different from the other ppGalNAc-T isoforms. To evaluate the expression of ppGalNAc-T4, the *GALNTs* mRNA expression profile across multiple tumor samples and paired normal tissues was checked using online data sets (GEPIA) (Figs. S1, *A–B*). *GALNT4* was one of *GALNTs*, which showed differential expression level in breast cancer. According to molecular features, breast cancer can be classified into some major subtypes: luminal (Lum A and Lum B), human epidermal growth factor 2 (HER2)-enriched, basal-like (BL), and claudin-low (CL) (39). Lum A and Lum B subtypes typically have a better prognosis, while BL tumors have a poor 5-year survival rate with high metastasis and recurrence (40). To investigate whether ppGalNAc-T4 is functionally involved in regulating the aggressiveness of breast cancer cells, we used online data sets (GEPIA and Kaplan–Meier) to validate *GALNT4* (gene name of ppGalNAc-T4) expression (41, 42). The results showed that in Lum subtype, *GALNT4* was significantly upregulated in breast tumors compared with that in the normal breast tissues. No differential expression of *GALNT4* was found between Lum A and B. In the BL subtype, *GALNT4* expression showed no significant difference between breast tumor and normal tissues (Fig. 1*A*). A high probability of recurrence-free survival (RFS) was correlated with a high *GALNT4* expression level, indicating the function of this gene in breast cancer progression (Fig. 1*B*). Further, we screened the expression of ppGalNAc-T4 in low-metastatic Lum subtype breast cancer cells (MCF7 and T47D) and mesenchymal-like highly invasive basal-like cells (MDA-MB-468, MDA-MB-453, MDA-MB-435, MDA-MB-231 and BT549) using real-time quantitative PCR (qPCR, Fig. 1*C*) and western blot analysis (Fig. 1*D*). In the seven cell lines used, endogenous ppGalNAc-T4 expression levels were relatively higher in Lum subtypes than in BL subtypes. The mRNA and protein expression levels of ppGalNAc-T4 in breast cancer cell lines correlated with the mRNA of online patient samples. These data suggested a possible connection between ppGalNAc-T4 and metastasis potential in breast cancer cells.

### ppGalNAc-T4 regulates breast cancer cell metastasis potential *via* TGF-β-induced EMT

**Figure 2:**
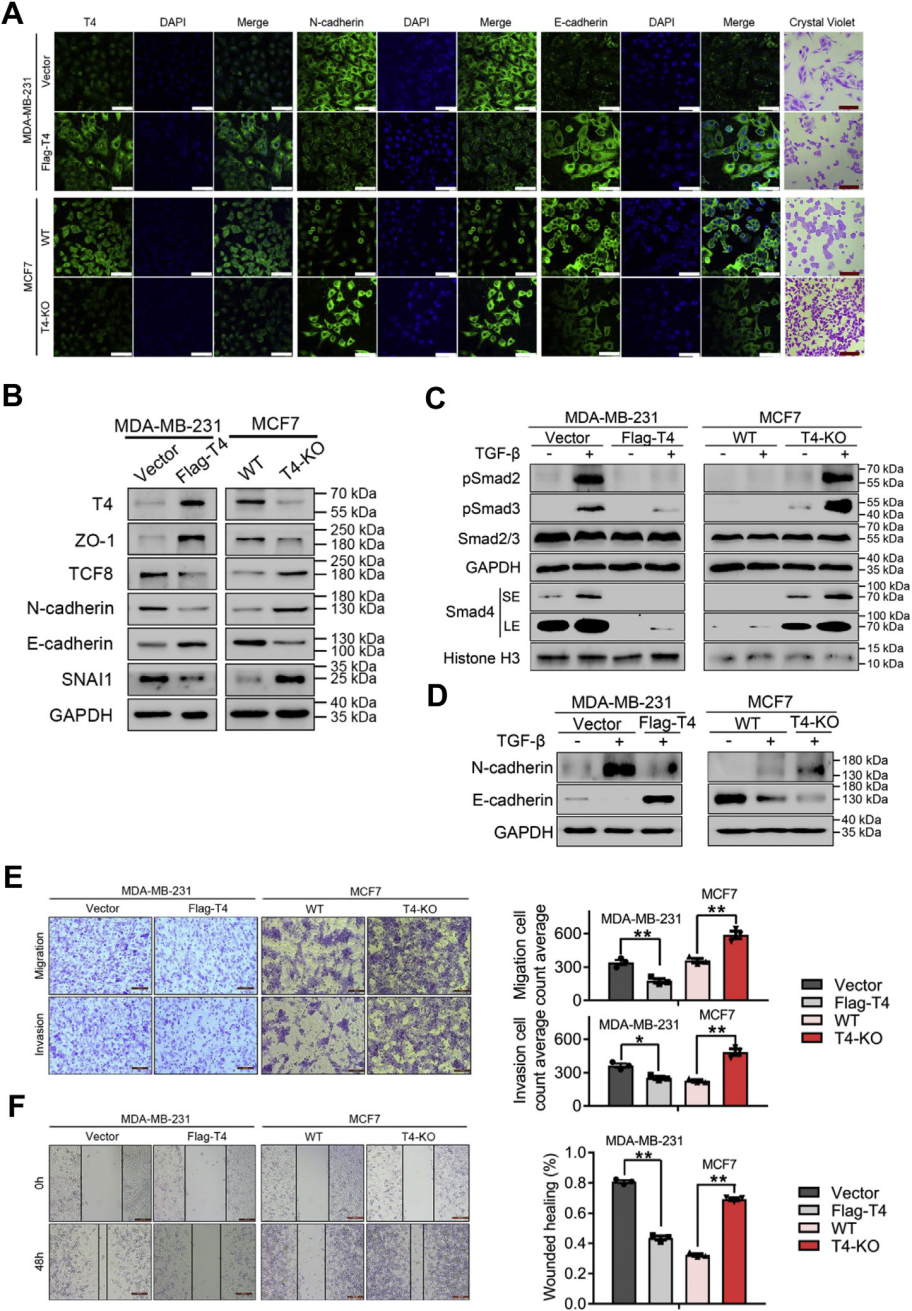
**ppGalNAc-T4 regulates breast cancer cell metastasis potential *via* TGF-β-induced EMT process.***A*, immunofluorescence staining was performed to detect protein expression of ppGalNAc-T4, N-cadherin and E-cadherin in MDA-MB-231 and MCF7 cells after ppGalNAc-T4 was regulated. Nuclei were counterstained with DAPI (white scale bar, 75 μm). Cell morphology was captured by crystal violet staining (*red* scale bar, 200 μm). *B*, ppGalNAc-T4 was upregulated in MDA-MB-231 cell by transient transfection and knocked out in MCF7 cell by CRISPR-Cas9. ppGalNAc-T4, ZO-1, TCF8, N-cadherin, E-cadherin, SNAI1, and GAPDH were detected by western blot. *C–D*, MDA-MB-231 and MCF7 cells were stimulated with TGF-β (5 ng/ml) overnight after ppGalNAc-T4 was regulated. EMT marker N-cadherin and E-cadherin were examined by western blot analysis. Phosphorylation levels of Smad2, Smad3 and accumulation of Smad4 in nucleus were measured by western blot analysis (SE, short exposure; LE, long exposure). GAPDH was used as the internal control and Histone H3 served as the nucleus protein loading control. *E–F*, Cell migratory and invasive ability of MDA-MB-231 and MCF7 cells after ppGalNAc-T4 was regulated was shown by transwell assay with and without Matrigel coating (scale bar, 100 μm) and wound healing assay (Scale bar, 200 μm). The data were obtained from three independent experiments, presented as mean ± SD. *P* values by paired *t*-test; ∗*p* < 0.05, ∗∗*p* < 0.01.

During breast tumor progression, metastasis is a major life-threatening event involving changes in many molecules. MDA-MB-231 (basal-like) and MCF7 (luminal type) are human breast adenocarcinoma epithelial cells with high and low migratory and invasive abilities, respectively. To define the role of ppGalNAc-T4 in breast cancer, we upregulated ppGalNAc-T4 in MDA-MB-231 cells using an overexpression plasmid and knocked out ppGalNAc-T4 in MCF7 cells using CRISPR/Cas9-mediated genome editing (Fig. S2). As shown by crystal violet staining, cells exhibited an epithelial morphology after ppGalNAc-T4 overexpression and a mesenchymal morphology after ppGalNAc-T4 knockout (Fig. 2*A*). These cell morphological changes indicated that ppGalNAc-T4 might participate in EMT. Moreover, ppGalNAc-T4 overexpression increased the epithelial markers ZO-1 and E-cadherin and reduced the mesenchymal marker N-cadherin at both the transcriptional and protein levels. In addition, TCF8 and SNAI1, which are EMT-inducing transcription factors, were reduced (Fig. S3*A* and Fig. 2*B*). Consistently, ppGalNAc-T4 knockout-induced ZO-1 and E-cadherin decreased, while N-cadherin, TCF8, and SNAI1 increased (Fig. S3*B* and Fig. 2*B*). The expression of N-cadherin and E-cadherin was further confirmed by immunofluorescence staining (IFC, Fig. 2*A*). These EMT-related factors exhibited almost the same expression level in ppGalNAc-T4-overexpression MDA-MB-231 cells and wild-type MCF7 cells, which indicated a regulatory role of ppGalNAc-T4 between the transition of epithelial and mesenchymal status. To verify the biological function of ppGalNAc-T4 in cell metastasis potential, we performed transwell and wound healing assays (Fig. 2, *E*–*F*). In MDA-MB-231 cells, cell motility was impaired, and cell junctions tightened after ppGalNAc-T4 was upregulated. The opposite results were obtained in MCF7 cells when ppGalNAc-T4 was knocked out.

As a master regulator of the EMT process (43, 44), TGF-β signaling converges in the nucleus to reprogram a set of target transcription factors (45), among which TCF8 and SNAI1 were regulated by ppGalNAc-T4 in the above results. To further confirm the role of ppGalNAc-T4 in cell metastasis potential, we treated MDA-MB-231 and MCF7 cells with TGF-β followed by detection of the EMT process and TGF-β signaling activity. Upon treatment with TGF-β, the phosphorylation levels of Smad2 and Smad3 (pSmad2 and pSmad3) as well as the nuclear accumulation of Smad4 were decreased when ppGalNAc-T4 was overexpressed in MDA-MB-231 cells. In contrast, the pSmad2 and pSmad3 levels and nuclear accumulation of Smad4 were elevated when ppGalNAc-T4 was knocked out in MCF7 cells (Fig. 2*C*). In addition, the TGF-β-induced EMT process was impaired by overexpression of ppGalNAc-T4, and these responses were severely elevated by knockout of ppGalNAc-T4 (Fig. 2*D*). Together, these data suggest that ppGalNAc-T4 is critical for TGF-β/Smad signaling-induced EMT in breast cancer cells.

### ppGalNAc-T4 interacts with TGF-β receptors in cells

**Figure 3:**
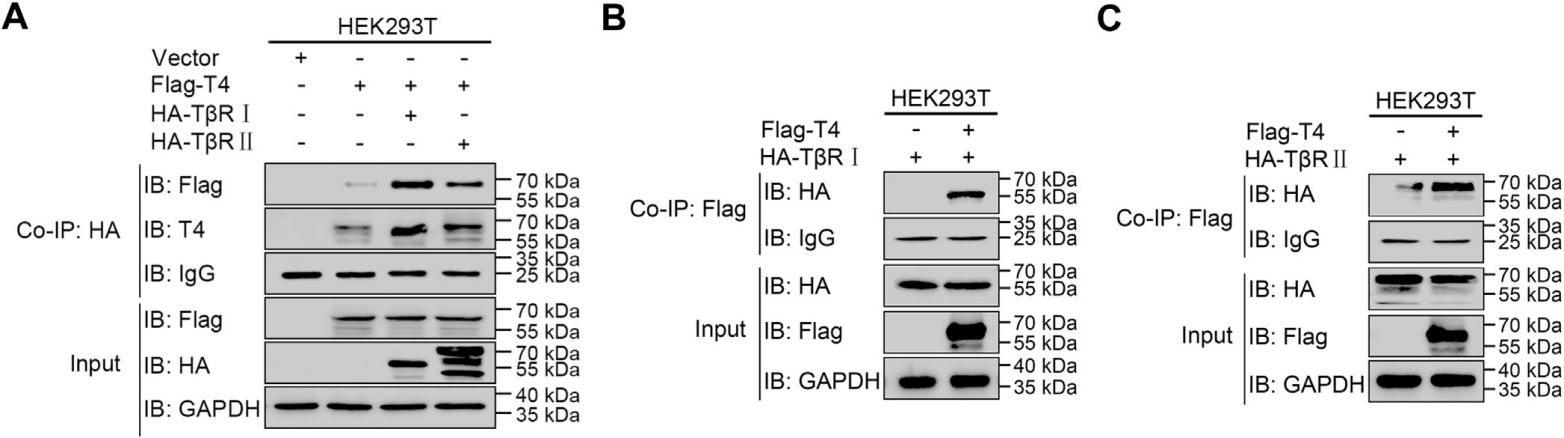
**ppGalNAc-T4 interacts with TGF-β receptors in cells.***A*, flag-T4, HA-TβR Ⅰ, HA-TβR Ⅱ, and empty vector were transfected into HEK-293T cell. The cell lysates were coimmunoprecipitated with anti-HA magnetic beads followed by immunoblotting using anti-Flag and anti-ppGalNAc-T4 antibodies. Co-IP: anti-HA, IB: anti-Flag, anti-T4. HA-TβR Ⅰ (*B*) or HA-TβR Ⅱ (*C*) and Flag-T4 were transfected into HEK-293T cell. The cell lysates were coimmunoprecipitated with anti-Flag magnetic beads followed by immunoblotting using anti-HA antibody. Co-IP: anti-Flag, IB: anti-HA. The inputs and immunoprecipitates were examined by western blot using corresponding antibodies.

Given the importance of ppGalNAc-T4 in TGF-β signaling activity, whether ppGalNAc-T4 interacts with TGF-β type Ⅰ and R Ⅱ receptors (TβR Ⅰ and TβR Ⅱ) was detected. The exogenously expressed Flag-T4 (T4 is short for ppGalNAc-T4) and HA-TβR Ⅰ or R Ⅱ in HEK-293T cells were examined by coimmunoprecipitation (co-IP) assays to validate the T4/TβR Ⅰ and T4/TβR Ⅱ interaction. Flag-T4 was found to bind to HA-TβR Ⅰ and HA-TβR Ⅱ when the cell lysates were immunoprecipitated with anti-HA magnetic beads and immunoblotted with anti-Flag antibody as well as ppGalNAc-T4 antibody (Fig. 3*A*). Additionally, both HA-TβR Ⅰ (Fig. 3*B*) and HA-TβR Ⅱ (Fig. 3*C*) were observed to interact with Flag-T4 when anti-Flag magnetic beads were used. These results indicated that ppGalNAc-T4 associates with TβR Ⅰ and Ⅱ in living cells, which could open up the possibility for the O-GalNAcylation of TGF-β receptors.

### ppGalNAc-T4-dependent O-GalNAcylation disturbs the assembly of TGF-β receptors

**Figure 4:**
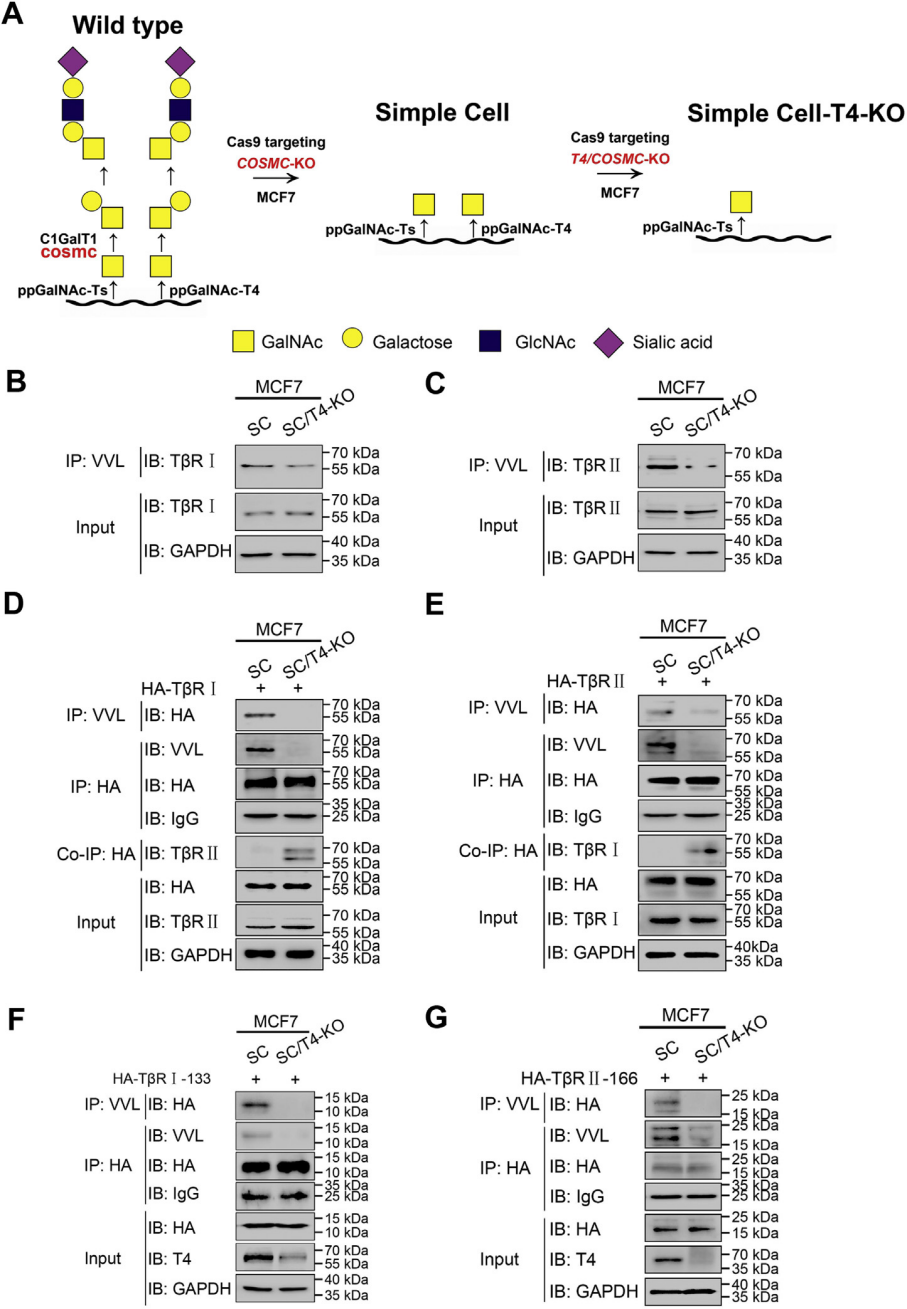
**ppGalNAc-T4 dependent O-GalNAcylation disturbs the assembly of TGF-β receptors.***A*, schematic diagram of O-GalNAcylation in wild-type cell, SimpleCell (COSMC-KO), and SimpleCell-T4-KO (T4/COSMC-KO). *B–C* MCF7-SC and MCF7-SC-T4-KO cell lysates were denatured and immunoprecipitated with agarose-bound VVL followed by immunoblotting with anti-TβR Ⅰ or anti-TβR Ⅱ antibody. IP: anti-VVL, IB: anti-TβR Ⅰ or anti-TβR Ⅱ. *D*, MCF7-SC and MCF7-SC-T4-KO cells were transfected with HA-TβR Ⅰ. The cell lysates were divided into two and one was denatured and immunoprecipitated with agarose-bound VVL and anti-HA magnetic beads followed by immunoblotting with anti-HA antibody and VVL. Another one was immunoprecipitated with anti-HA magnetic beads followed by immunoblotting with TβR Ⅱ antibody. IP: anti-VVL, IB:anti-HA; IP:anti-HA, IB: anti-VVL; Co-IP: anti-HA, IB: anti-TβR Ⅱ. (E) MCF7-SC and MCF7-SC-T4-KO cells were transfected with HA-TβR Ⅱ. The cell lysates were divided into two and one was denatured and immunoprecipitated with agarose-bound VVL and anti-HA magnetic beads followed by immunoblotting with anti-HA antibody and VVL. Another one was immunoprecipitated with anti-HA magnetic beads followed by immunoblotting with TβR Ⅰ antibody. IP: anti-VVL, IB: anti-HA; IP: anti-HA, IB: anti-VVL; Co-IP: anti-HA, IB: anti-TβR Ⅰ. F*–G*, MCF7-SC and MCF7-SC-T4-KO cells were transfected with extracellular domain of TβR Ⅰ, HA-TβR Ⅰ-133 or extracellular domain of TβR Ⅱ, HA-TβR Ⅱ-166. The cell lysates were denatured and immunoprecipitated using agarose-bound VVL and anti-HA magnetic beads followed by immunoblotting with anti-HA antibody and VVL. IP: anti-VVL, IB: anti-HA; IP: anti-HA, IB: anti-VVL.

TGF-β signal transduction occurs *via* the interaction between TGF-β ligands and the heteromeric complex of TβR Ⅰ and TβR Ⅱ. N-linked glycosylation has been reported to regulate the binding activity of TGF-β ligands with receptors (46, 47). Since ppGalNAc-T4 obviously affected TGF-β/Smad signaling, we examined whether these two TGF-β receptors are modified by ppGalNAc-T4-catalyzed O-GalNAcylation. The protein-specific chaperone COSMC is essential for Tn antigen elongation, which results in the abundance and diversity of GalNAc-type O-glycosylation; thus we generated COSMC knockout (SimpleCell, SC) (48) and COSMC/T4 double-knockout (SC-T4-KO) MCF7 cell lines (Figs. S4–S5) to prevent Tn antigen extension and simplify the O-GalNAc glycans on substrate proteins (Fig. 4*A*).

To identify the O-GalNAcylation of TβR Ⅰ and R Ⅱ, we performed immunoprecipitation under denaturing conditions using *Vicia villosa* lectin (VVL, recognizes terminal N-acetylgalactosamine residues linked to serine or threonine in a polypeptide, Tn antigen) agarose beads. The immunoprecipitated products were detected by TβR Ⅰ or R Ⅱ antibodies. Bands corresponding to O-GalNAcylated TβR Ⅰ (Fig. 4*B*) and R Ⅱ (Fig. 4*C*) were observed in MCF7-SC, indicating that endogenous TβR Ⅰ and R Ⅱ were O-GalNAcylated. We then transfected MCF7-SC and MCF7-SC-T4-KO cells with plasmids expressing HA-tagged TGF-β receptors and performed immunoprecipitation VVL-agarose beads and anti-HA magnetic beads (Figs. 4, *D*–*E*). Substantially, O-GalNAc modification on exogenous TβR Ⅰ and R Ⅱ was diminished or abolished after ppGalNAc-T4 knockout in the MCF7-SC-T4-KO cell line, suggesting the catalytic function of ppGalNAc-T4 in TβR Ⅰ and R Ⅱ O-GalNAcylation. Additionally, a co-IP assay was performed to examine the TβR Ⅰ and R Ⅱ interaction. We found that reduced O-GalNAcylation of TβR Ⅰ and R Ⅱ enhanced the dimerization between them. ppGalNAc-T4 did not affect TβR Ⅰ and R Ⅱ expression, but the O-GalNAcylation and the interaction between them. Similar detections were performed in HEK-293T cell line. TβR Ⅰ and R Ⅱ were also observed to be O-GalNAcylated by ppGalNAc-T4 (Figs. S6, *A–B*). These data distinctly demonstrated that TGF-β receptors are modified by O-GalNAcylation in a ppGalNAc-T4-dependent manner, and the TGF-β receptor complex is disturbed by this modification.

Studies have shown that O-GalNAc-type modified glycoproteins are produced as membrane and secretory proteins (49, 50, 51). Given that TβR Ⅰ and TβR Ⅱ have been proven to be O-GalNAcylated by ppGalNAc-T4, HA-tagged extracellular domains of both TGF-β receptors (aa 1–133 for TβR Ⅰ and aa 1–166 for TβR Ⅱ) were used to determine whether ppGalNAc-T4 induces O-GalNAcylation of the extracellular domains. The extracellular domains of TGF-β receptors were exogenously expressed in MCF7-SC and MCF7-SC-T4-KO cells. Duplex immunoprecipitation under denaturing conditions with VVL-agarose beads and anti-HA magnetic beads revealed that modified amounts of TβR Ⅰ and R Ⅱ were significantly reduced accompanied by ppGalNAc-T4 knockout (Figs. 4, *F*–*G*). The experiments in HEK-293T cells gave similar results (Figs. S6, *C–D*), indicating the participation of ppGalNAc-T4 in the extracellular domain O-GalNAcylation.

### TGF-β type Ⅱ receptor is an *in vitro* substrate of ppGalNAc-T4

**Figure 5:**
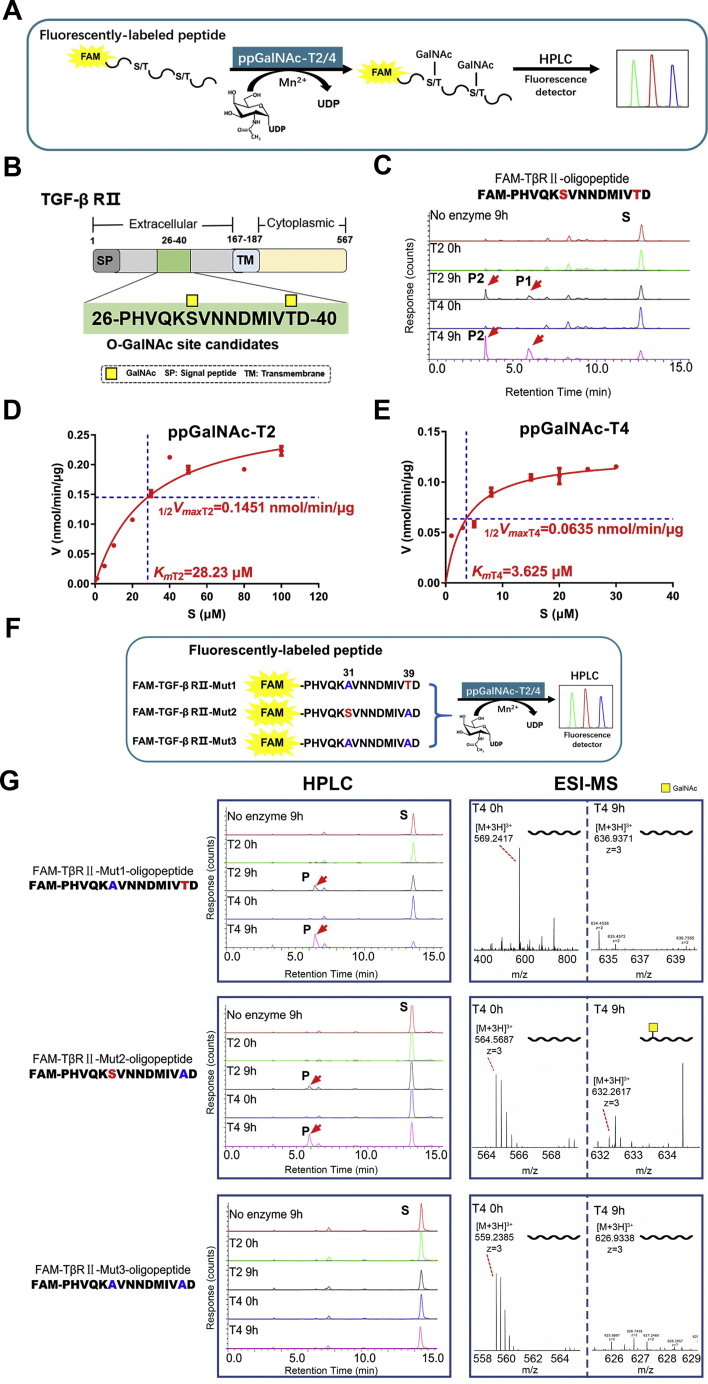
**TGF-β type Ⅱ receptor is an *in vitro* substrate of ppGalNAc-T4 at Ser31.***A*, schematic diagram of the process of the O-GalNAcylation of the FAM-labeled peptide catalyzed by ppGalNAc-T2 or -T4 *in vitro*. *B*, protein domain organization of TβR Ⅱ and possible O-GalNAcylation sites. *C*, products derived from ppGalNAc-T2 or -T4 reaction of TβR Ⅱ peptides with possible O-GalNAcylation sites were analyzed by HPLC. S, P1, and P2 correspond to substrate, monoglycosylated products, and diglycosylated products, respectively. D*–E* The *K*_*m*_ and *V*_*max*_ of ppGalNAc-T2 or -T4 were calculated by nonlinear fitting to Michaelis–Menten equation following plotting the results of enzymatic activity obtained with different concentrations of the substrate of FAM-PHVQKSVNNDMIVTD. *F*, synthesis of the TβR Ⅱ short peptides with possible O-GalNAcylation sites and sites mutations (Mut1, S31 A; Mut2, T39 A; Mut3, S31 A/T39 A). *G*, products derived from ppGalNAc-T2 or -T4 reaction of the TβR Ⅱ short peptides with possible O-GalNAcylation sites and sites mutations were analysis by HPLC and MS. S, P correspond to substrate and monoglycosylated products, respectively.

To further investigate the specific mechanism of ppGalNAc-T4-catalyzed O-GalNAcylation on TGF-β receptors, we used an HPLC-based *in vitro* O-GalNAc enzymatic detection system. By comparing the putative O-GalNAcylated peptide sequences reported by Steentoft in the human O-GalNAc glycoproteome and the predicted results from a publicly available database NetGlyc 4.0 Server (http://www.cbs.dtu.dk/services/NetOGlyc/), we selected multiple candidate sequences covering most of the TβR Ⅰ and R Ⅱ extracellular domains containing putative O-GalNAcylation sites for probing the modification (Figs. S7, *A–B*). These fluorescently labeled synthetic peptides act as acceptors of O-GalNAcylation (Fig. 5*A*) (52). It was proven that the glycosylation of previously reported acceptor peptides by ppGalNAc-T4 requires prior glycosylation by other ppGalNAc-Ts with different substrate specificities. ppGalNAc-T2 (T2), which is broadly expressed in various human tissues and uses naked peptides as an O-GalNAc acceptor substrate, was employed as a control. Flag-tagged T2 and T4 were reintroduced into HEK293 T cells, and the ppGalNAc-T2 and ppGalNAc-T4 recombinant enzymes were purified from the supernatant by immunoprecipitation with anti-FLAG affinity beads (Fig. S7*C*).

Intriguingly, in a ppGalNAc-T4-containing HPLC assay using naked FAM-TβR Ⅱ peptide as the acceptor (aa 26–40, PHVQK**S**VNNDMIV**T**D), two new peaks (P1 and P2) with advanced retention time at 3.5 and 6.0 min were observed (Figs. 5, *B*–*C*) compared with the blank control reaction, indicating that the candidate peptide from extracellular domain of TβR Ⅱ could be O-GalNAcylated by ppGalNAc-T4. The retention time of other candidate peptides did not change after the enzymatic reaction (Figs. S7, *D–I*), revealing that ppGalNAc-T4 had no detectable activity with these peptides *in vitro*. Since both serine 31 (Ser31) and threonine 39 (Thr39) were predicted as the O-GalNAcylation sites on the TβR Ⅱ peptide, we supposed that P1 represented the glycosylation products with just one GalNAc residue at either Ser31 or Thr39, while P2 represented the glycosylation products with two GalNAc residues at both sites. In good agreement with this result, these O-GalNAcylated products were also obtained in the ppGalNAc-T2 enzymatic reaction. These data suggested that a naked peptide sequence from the TGF-β type Ⅱ receptor is a new *in vitro* substrate of ppGalNAc-T4 and that two potential glycosylation sites, Ser31 and Thr39, exist in the TβR Ⅱ peptide.

Notably, kinetic analysis of ppGalNAc-T2 (Fig. 5*D*) and ppGalNAc-T4 (Fig. 5*E*) activity with naked TβR Ⅱ peptide was performed. Kinetic parameters (*V*_*max*_ and *K*_*m*_) were determined by nonlinear fitting to the Michaelis–Menten equation, and the results showed that the *V*_*max*_ and *K*_*m*_ values of ppGalNAc-T2 were higher than those of ppGalNAc-T4 for the FAM-TβR Ⅱ peptide. These data demonstrated that ppGalNAc-T4 had a higher affinity toward the FAM-TβR Ⅱ peptide than ppGalNAc-T2. ppGalNAc-T4 may play a major role in modifying TβR Ⅱ in living cells.

### Ser31 of TβR Ⅱ was identified as the O-GalNAcylation site by ppGalNAc-T4

To further confirm the authenticity of TβR Ⅱ O-GalNAcylation sites, we synthesized a TβR Ⅱ peptide in which serine and/or threonine residues were mutated to alanine (A). Sequences of the mutants are shown in Figure 5*F* (Mut1, S31 A; Mut2, T39 A; Mut3, S31 A/T39 A). HPLC and ESI-MS were performed to analyze the O-GalNAcylation sites (Fig. 5*G*). From the *in vitro* ppGalNAc-T2/-T4 enzymatic reaction assay, P was detected in both S31 A and T39 A single mutants, suggesting that one GalNAc residue could be added at Thr39 in Mut1 and Ser31 in Mut2 in the HPLC assay. ESI-MS showed a mass increase of one GalNAc residue (m/z +67.6930 Da) in Mut2, suggesting that ppGalNAc-T4 catalyzed the transfer of GalNAc from UDP-GalNAc to Ser31. There was no mass change in Mut1 and double-mutant Mut3 peptide, indicating that Thr39 was not glycosylated, despite the shift in retention time of HPLC.

**Figure 6:**
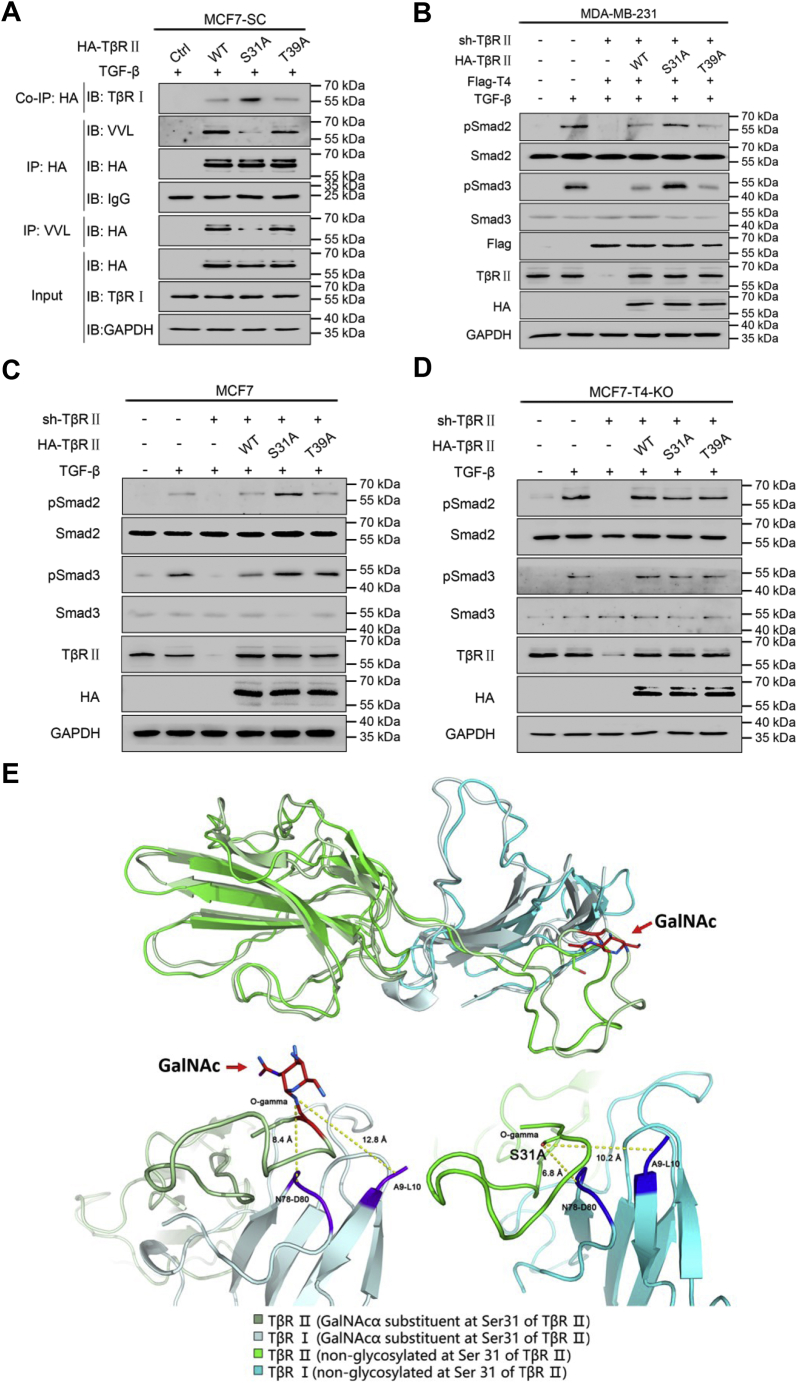
**ppGalNAc-T4 modulates TGF-β signaling *via* catalyzing O-GalNAcylation of TGF-β type Ⅱ receptor at Ser31.***A*, MCF7-SC transfected with WT or mutant HA-TβR Ⅱ was incubated with TGF-β. The cell lysates were treated as in Figure 4*C*. Co-IP: anti-HA, IB: anti-TβR Ⅰ; IP: anti-HA, IB: anti-VVL; IP: anti-VVL, IB: anti-HA. *B*–*D*, MDA-MB-231, MCF7, and MCF7-T4-KO cells were transfected with sh-TβR Ⅱ, WT or mutant HA-TβR Ⅱ (as well as Flag-T4 in MDA-MB-231 cells) with incubation of TGF-β. Phosphorylation levels of Smad2 and Smad3 were measured by western blot analysis. GAPDH was used as the internal control. *E*, the representative structures were superimposed with the overall C-alpha RMSD (root mean square deviation) 2.738 Å. Ser-31 and O-GalNAc residues were depicted in sticks, and the *yellow dash lines* show the collective variables CV1 (distance-1, distance between O-gamma atom of Ser31 in TβR Ⅱ and the center of mass of TβR Ⅰ9–10) and CV2 (distance-2, distance between O-gamma atom of Ser-31 in TβR Ⅱ and the center of mass of TβR Ⅰ 78–80) in the representative structures of O-GalNAcylated and nonglycosylated systems. The O-GalNAcylated (GalNAcα) substituent increased the distances between two proteins and would cause the global conformational change.

Since HPLC and ESI-MS provided two potential glycosylation sites of TβR Ⅱ, we generated single site mutated TβR Ⅱ constructs, HA-TβR Ⅱ-S31 A and HA-TβR Ⅱ-T39 A, for further confirmation. Wild-type (WT) and mutated constructs were then transfected into MCF7-SCs, and their O-GalNAcylation levels were determined. Interestingly, the S31 A single mutant exhibited a decreased O-GalNAcylation level compared with the WT, while there was no distinct modification change between the T39 A mutant and the WT. These results indicated that Ser31, but not Thr39, was the major O-GalNAcylation site for TβR Ⅱ in breast cancer cells (Fig. 6*A*).

### ppGalNAc-T4 modulates TGF-β signaling by catalyzing O-GalNAcylation of the TGF-β type Ⅱ receptor at Ser31

We then verified the participation of the O-GalNAcylation site of TβR Ⅱ in TGF-β signaling in breast cancer cells. Additionally, the Co-IP results in Figure 6*A* showed that the S31 A mutant, but not T39 A, elevated the association between TβR Ⅰ and TβR Ⅱ, suggesting an attenuating role for Ser31 O-GalNAcylation in TGF-β signaling. In MDA-MB-231 and MCF7 cells (Figs. 6, *B*–*C*), shRNA targeting TβR Ⅱ was used to silence endogenous TβR Ⅱ expression, which showed high efficiency and TβR Ⅱ was almost not to be seen. Then WT, Mut-S31 A, and Mut-T39 A groups were used resulting in rescuing TβR Ⅱ expression. Under TGF-β treatment, the levels of pSmad2 and pSmad3 were elevated in the S31 A mutant rescued group, whereas Smad2 and Smad3 phosphorylation in the WT and T39 A groups showed similarly low levels, suggesting that TGF-β signaling was activated after reducing the O-GalNAcylation of TβR Ⅱ at Ser31. In MCF7-T4-KO cells (Fig. 6*D*), TGF-β signaling unchanged, exhibiting the specific role of ppGalNAc-T4 in the regulatory process.

To further probe how O-GalNAcylation prevents the formation of the TβR Ⅰ and TβR Ⅱ complexes, we performed structure modeling, molecular dynamics simulation, and conformation landscape analysis. Fig. S8*A* shows three principal component (PC1, PC2, PC3) conformational changes upon TβR Ⅰ and TβR Ⅱ binding, and the motion is shown in Movie S1–S6. The distance of two critical contact pairs between the TβR Ⅰ (center of mass of TβR Ⅰ A9-L10 and N78-D80) and TβR Ⅱ (oxygen gamma atom of Ser31) complex was treated as the collective variable to compute the conformational landscape (Fig. 6*E*), which revealed the significant difference between the WT system and the modified system. The O-GalNAcylated (GalNAcα) chain substituent at Ser31 of TβR Ⅱ altered the landscape globally and generated two free energy minima, corresponding to much longer distances between TβR Ⅰ and GalNAcα substituent at Ser31 of TβR Ⅱ relative to that of nonglycosylated TβR Ⅱ (Fig. 6*E* and Fig. S8*B*). The O-GalNAcylated (GalNAcα) substituent increased the distances between the two proteins and caused a global conformational change. The interface areas between the two receptor complexes decreased after the GalNAcα substituent at Ser31 of TβR Ⅱ (Fig. S8*C*). Altogether, ppGalNAc-T4-dependent O-GalNAcylation of the TGF-β type Ⅱ receptor at Ser31 results in dissociation of the TβR Ⅰ and TβR Ⅱ complex and regulates TGF-β signaling in breast cancer cells.

## Discussion

**Figure 7:**
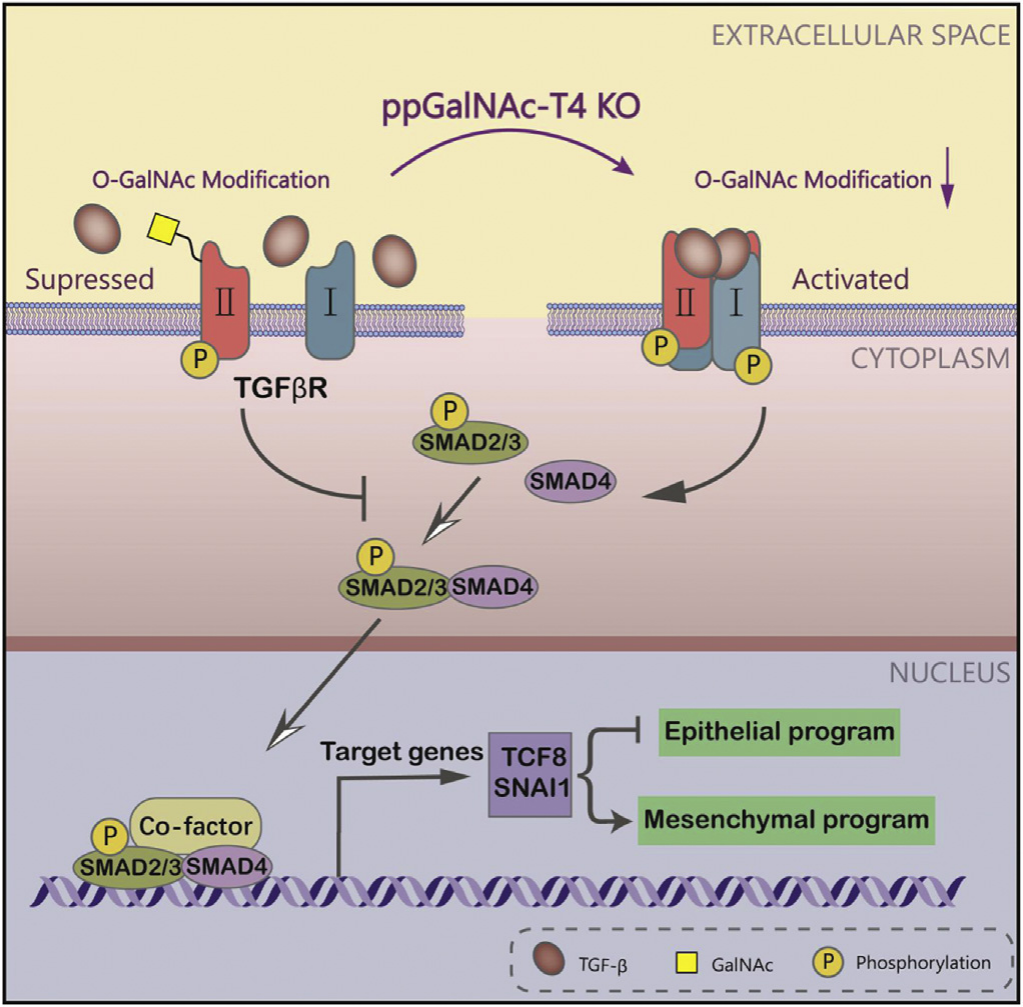
**Schematic of proposed function and mechanism of ppGalNAc-T4 in breast cancer progression.** Loss of ppGalNAc-T4 results in the decrease O-GalNAcylation of TβR Ⅰ and R Ⅱ, which promotes the dimerization of them and activates TGF-β signaling pathway. These events facilitate the transformation of the epithelial phenotypes toward mesenchymal features, with increased migration and invasion abilities in human breast cancer cells. ppGalNAc-T4 catalyzes TGF-β receptors O-GalNAc modification regulating breast carcinoma cells metastasis potential *via* EMT process.

Herein, we define a critical role of O-GalNAc glycosylation in human breast cancer cell metastasis potential. We demonstrated that O-GalNAc glycosylation of TGF-β type Ⅰ and R Ⅱ receptors is dependent on ppGalNAc-T4. Loss of ppGalNAc-T4 resulted in decreased O-GalNAcylation of TβR Ⅰ and R Ⅱ, which promoted their dimerization and activated the TGF-β signaling pathway (Fig. 7). These events facilitated the transformation of the epithelial phenotypes toward mesenchymal features, with increased migration and invasion of human breast cancer cells. Notably, we identified a naked peptide from the extracellular domain of TβR Ⅱ that could be O-GalNAcylated by ppGalNAc-T4 *in vitro*. Ser31 was further demonstrated as the O-GalNAcylation site *via* both *in vitro* and in cell assays. Together, our study reveals a novel mechanism of ppGalNAc-T4-catalyzed TGF-β receptor O-GalNAcyaltion that suppresses breast carcinoma cell migration and invasion *via* the EMT process.

Abnormal O-GalNAc initiation is associated with cancer and several human disorders and has a relatively simple structure composed of N-acetyl-D-galactosamine (53). Aberrant expression of initiating ppGalNAc-Ts would result in such truncated glycan and disrupted protein structures and biological functions. In colorectal cancer (CRC), ppGalNAc-T4 expression was significantly reduced with tumor progression (33). In addition, Liu *et al*. showed the negative impact of ppGalNAc-T4 expression on the malignant transformation of HCC (54). In this study, a different expression level of ppGalNAc-T4 was observed in normal and breast tumor tissues, and high expression of ppGalNAc-T4 was associated with better prognosis (Figs. 1, *A*–*B*). In normal breast tissue or cell lines, ppGalNAc-T4 expression might provide a basic but necessary function to maintain regular physiological activities, while in the carcinoma environment, ppGalNAc-T4 may act as a cancer inhibitor by modifying certain specific substrate proteins, such as TβR Ⅱ, in this study. This specific O-GalNAcylation might influence the structure and function of substrate proteins to promote cancer cell behaviors. To clarify the complicated mechanisms of breast cancer metastasis, researchers must further study the specific substrates of ppGalNAc-T4 and other enzymes. Although previous findings have revealed the role of ppGalNAc-T4 in tumor progression, rare ppGalNAc-T4-specific substrates were reported to date. Liu has reported that GALNT4 could modify the O-linked glycosylation and regulate the activity of EGFR (34); Pratt has reported that naked EA2 peptide is a positive substrate for ppGalNAc-T4 (35); Bennett has reported that GalNAc-T4 plays important roles in glycosylation of PSGL-1 and MUC1 (36). These results indicated that ppGalNAc-T4 is one of the isoforms, which prefers to catalyze GalNAcylated substrates by other ppGalNAc-Ts.

In our study, the SimpleCell strategy was employed to investigate the biological function of ppGalNAc-T4. This strategy uses a genetically engineered cell line in which the *COSMC* gene, the molecular chaperone for T-synthase, is knocked out (KO) for homogeneous Tn and/or Sialyl-Tn (STn) O-glycan structures accumulation. This strategy enriched unmodified O-GalNAcylated structure (Tn), which can be recognized and isolated by VVL lectin and simplifies the substrates analytical steps. If necessary, sialic acids can be removed by neuraminidase treatment to further enrich Tn structure. In this study, TGF-β receptors can be recognized and pulled down by VVL lectin without treatment of neuraminidase, indicating that at least a part of TGF-β receptors in MCF7-SC cells are O-GalNAcylated. We cannot rule out that TGF-β receptors contain Sialyl-Tn structure and further study is needed to confirm it. In addition, the expression of other ten ppGalNAc-Ts (T2, T3, T6, T11, T14, and T16, which have been reported involved in TGF-β receptors or signaling; T12, which is clustered in the same subgroup with T4 in family evolutionary tree) was determined to be unchanged with ppGalNAc-T4 knockout (Fig. S9), indicating the applicability of these cells for ppGalNAc-T4-specific research. From the results of immunoprecipitation assays in MCF7-SC and MCF7-SC/T4-KO cell lines, we confirmed that ppGalNAc-T4 regulates the O-GalNAc modification of TβR Ⅰ and R Ⅱ.

TβR Ⅰ and R Ⅱ are transmembrane proteins that act as receptors of TGF-β signaling, which is hyperactivated and glycosylated in advanced cancers (55). Previously, it was confirmed that TβR Ⅱ has N-linked glycosylation at Asn70 and 94, which controls its transportation to the cell surface membrane, followed by an impaired TGF-β-mediated signaling pathway (46). In cells undergoing EMT, ligand-bound TβR Ⅱ directly induces TβR Ⅰ to activate Smad2/3 and subsequently EMT-related transcription factors. Our data showed that ppGalNAc-T4 catalyzes TβR Ⅰ and R Ⅱ O-GalNAc glycosylation, which inhibits heterodimer combination and thus suppresses downstream signaling-induced EMT. By using the SimpleCell strategy, we demonstrated that TβR Ⅰ and R Ⅱ are O-GalNAcylated by ppGalNAc-T4 and subsequently influence TGF-β signaling activity. Loss of ppGalNAc-T4 resulted in elevated pSmad2/3 and nuclear Smad4 accumulation in breast cancer cells (Fig. 2*C*). Correspondingly, ppGalNAc-T4 correlates negatively with N-cadherin, but positively with E-cadherin. Increased TCF8 and SNAI1-induced EMT, which resulted in enhanced migratory and invasive capabilities, was observed in breast cancer cells with ppGalNAc-T4 knockdown. In further cell mutagenesis assays, rescuing of the mutant O-GalNAcylation site (S31 A) of TβR Ⅱ caused weakened O-GalNAc modification, which promoted TGF-β signaling activity and subsequently influenced cell metastasis potential in breast cancer. Our data identified a key role for ppGalNAc-T4 in human breast cancer cell metastasis potential by O-GalNAc modifying its target proteins TβR Ⅰ and R Ⅱ. However, the precise mechanism of O-GalNAcylation on TGF-β receptors remains to be elucidated. Here, we provide several potential hypotheses. First, the modification may affect protein folding, resulting in accumulation in the perinuclear region, which inhibits ligand binding. Alternatively, O-GalNAcylation might change the conformation of TβR Ⅰ and R Ⅱ on the cell surface, which blocks the interaction and association between them or impairs ligand–receptor binding affinity.

To further elucidate about how ppGalNAc-T4 affected TβR Ⅰ and R Ⅱ association, we carried out structural modeling, molecular dynamics simulation, and conformation landscape analysis (Fig. 6*E*). The O-GalNAcylated (GalNAcα) chain substituent at Ser31 of TβR Ⅱ altered the landscape globally, and the distances between TβR Ⅰ and the O-GalNAcylated (GalNAcα) chain substituent at Ser31 of TβR Ⅱ became significantly longer than those of the wild-type of TβR Ⅱ (Fig. S8*B*). Furthermore, the interface areas between the two receptor complexes decreased after the GalNAcα substituent at Ser31 of TβR Ⅱ (Fig. S8*C*). This may be one of the mechanisms by which ppGalNAc-T4 regulates TGF-β-induced EMT in breast cancer cells.

Since ppGalNAc-Ts comprise a large evolutionarily conserved family of O-GalNAc glycosyltransferases, the enzymes in this family show distinct acceptor substrate specificities and different expression patterns in tissue distribution (56,57). ppGalNAc-T4 is unique in that it is the only GalNAc-transferase isoform identified so far that is indispensable *in vitro* to produce full O-glycan occupancy in the tandem repeat of MUC1, which complements ppGalNAc-T1, -T2, and -T3 function (36). Research has shown that ppGalNAc-T4 exhibited clear activity toward previously glycosylated peptides catalyzed by other GalNAc-transferase isoforms (38). Although this enzyme can use naked EA2 as a substrate, the catalytic activity is low (35). In our study, the peptide from TβR Ⅱ was identified as a new naked peptide substrate of ppGalNAc-T4, which is an improvement over previous knowledge on ppGalNAc-T4 substrate specificity. It has been reported that ppGalNAc-T4 transfers GalNAc to two sites in the glycosylated-MUC1 sequence TAPPAHGVTSAPDTRPAPGSTAPPA. There are no similarities in a BLAST comparison between the sequence besides Ser31 of TβR Ⅱ and the MUC1-glycosylated peptide. The reason for this may be that the mechanism of recognition for naked or glycosylated peptide substrates might be different, and there is not much comparability between them. Further, reported peptide sequences of ppGalNAc-Ts substrates are very limited so far, and it would be inaccurate to determine the conservation sequence of ppGalNAc-Ts substrates based on limited data. A large amount of information about ppGalNAc-Ts substrate sequences is needed for precisely analyzing enzyme substrate specificity.

To determine whether there were nonspecific effects of the ppGalNAc-T4-catalyzed reaction, we used recombinant ppGalNAc-T2 as a positive control. In HPLC assays, the products of the ppGalNAc-T4-catalyzed reaction (P1 and P2 in Fig. 5*C*) had the same retention time as those of the ppGalNAc-T2-catalyzed reaction, suggesting that ppGalNAc-T4 targets the same sites on this peptide as ppGalNAc-T2. This result also eliminates potential nonspecific reactions of this peptide, such as aggregation. Our results provide a new substrate peptide for the ppGalNAc-T4 enzymatic reaction *in vitro* for further research. Although both isoform enzymes ppGalNAc-T2 and -T4 had catalytic activity with the TβR Ⅱ-derived peptide, ppGalNAc-T4 showed a higher affinity than ppGalNAc-T2 in kinetic properties. These data suggested that ppGalNAc-T4 is more selective for TβR Ⅱ than ppGalNAc-T2 *in living cells*. Although the function and expression of ppGalNAc-T2 have been widely demonstrated in cancer cells, other isoform enzymes of the ppGalNAc-Ts family, such as ppGalNAc-T4, might be more suitable and play a major role in catalyzing the O-GalNAc modification of TβR Ⅱ in certain tissues and cells.

To confirm the O-GalNAcylation sites of TβR Ⅱ, we performed single and double site mutagenesis assays by HPLC and MS analysis. O-GalNAc modification at Ser31 was proven by both HPLC and MS, while glycosylation at Thr39 was detected only by HPLC. In the mut1 (S31 A) HPLC assay (Fig. 5*G*), the new peak with an advanced retention time might not be a glycosylation product but a signal of a non-full-length peptide. The same results were observed with the wild-type peptide in Figure 5*C*. Furthermore, in the cell mutagenesis in cells assay, the O-GalNAcylation level of the S31 A mutant was strongly diminished, and the S31 A mutant, but not the T39 A mutant, altered the downstream TGF-β signaling. The results of the MS assay were in agreement with those of the cell assay, and Ser31 was identified as an O-GalNAcylated site of TβR Ⅱ by ppGalNAc-T4.

The immunoprecipitation in living cells indicated the O-GalNAcylation of TβR Ⅰ by ppGalNAc-T4 (Figs. 4, *B* and *D*), but *in vitro* enzymatic assays of peptides covering all possible glycosylation sites in the TβR Ⅰ extracellular domain showed that ppGalNAc-T4 had no activity (Fig. S7). We speculate that ppGalNAc-T4-catalyzed substrates of TβR Ⅰ may require prior glycosylation by other ppGalNAc-Ts. This may explain why naked TβR Ⅰ peptides are not catalyzed by ppGalNAc-T4 *in vitro*. Further investigations are needed to confirm the O-GalNAcylation site(s) in TβR Ⅰ.

In summary, we identified a posttranslational mechanism of TGF-β-induced EMT regulation through ppGalNAc-T4-dependent O-GalNAc glycosylation of TβR Ⅰ and R Ⅱ. Importantly, O-GalNAc at Ser31 in TβR Ⅱ is a key regulator in signaling transduction, and a new naked peptide sequence from TβR Ⅱ is supposed to be a substrate of ppGalNAc-T4. Further investigation will determine the identity of other modified proteins and their function in tumor progression. Together, our study proposes novel insights into the role of O-GalNAc glycosylation in cancer cell metastasis potential.

## Experimental procedures

### GALNT4 mRNA expression level and RFS (recurrence-free survival) analysis of breast cancer base on GALNT4

Gene Expression Profiling Interactive Analysis (GEPIA) (http://gepia.cancer-pku.cn) is a web-based tool to analyze RNA sequencing expression data based on The Cancer Genome Atlas (TCGA) and Genotype-Tissue Expression databases (41). The Kaplan–Meier plotter (http://kmplot.com/analysis/index.php?p=background) is an online survival analysis tool containing 6234 patients with breast cancer based on Gene Expression Omnibus (GEO), European Genome-phenome Archive (EGA) and TCGA (42). In the research, the expression level of *GALNT4* in different subtypes of breast cancer and the RFS analysis were obtained *via* GEPIA and Kaplan–Meier plotter.

### Cell culture

HEK293 T cells, human breast cancer cells MCF7, T47D, MDA-MB-468, MDA-MB-453, MDA-MB-435, MDA-MB-231, and BT549 cells lines were purchased from Type Culture Collection of the Chinese Academy of Sciences (Shanghai, China) and were used within 6 months from resuscitation. HEK293 T cells were propagated in high-glucose Dulbecco’s modified Eagle’s medium (DMEM) (Gibco, USA) with 10% fetal bovine serum (FBS) (Gibco, USA). T47D cells were cultured in RPMI 1640 (Gibco, USA) with 10% FBS. MCF7 was maintained in MEM (Gibco, USA) with 0.01 mg/ml insulin and 10% FBS. MDA-MB-468, MDA-MB-453, MDA-MB-435, and MDA-MB-231 were propagated in Leibovitz’s L15 Medium (Gibco, USA) with 10% FBS. BT549 was maintained in RPMI 1640 with 0.023U/ml insulin and 10% FBS. All medium was supplemented with 1% penicillin/streptomycin antibiotics (Gibco, USA). All cells were cultured in humidified incubator at 37 °C, in an atmosphere containing 5% CO_2_. To induce EMT, culture medium was supplemented with TGF-β1 (Sino Biological, China) to generate mesenchymal-like cells.

### Plasmids and cell transfection

A human full-length *GALNT4* cDNA was isolated and cloned into a p3×Flag-CMV vector. Full-length and extracellular domain of human *TGFBR1* and *TGFBR2*, full-length mutants including *TGFBR2-*S31 A *and TGFBR2-*T39 A, was isolated and cloned into pLvEGP-HA vector respectively. Target sequence of short hairpin RNA (shRNA) specific for *TGFBR2* is CCTGACTTGTTGCTAGTCATA (exon), which has been proven to effectively silence TβR Ⅱ expression. All plasmids were transfected into indicated cells using Lipofectamine 3000 (Invitrogen, Carlsbad, CA) following the manufacture’s manual.

### Western blot and lectin blot

Total protein extracts and western blot were performed as previously described (58). The following antibodies and lectins were obtained: anti-ppGalNAc-T4, anti-TβR Ⅰ from Abcam, USA; anti- TβR Ⅱ, anti-GAPDH, anti-ZO-1, anti-TCF8, anti-N-cadherin, anti-E-cadherin, anti-SNAI1, anti-pSmad2, anti-pSmad3, anti-Smad2/3, anti-Smad4, anti-Histone H3, anti-HA, anti-Flag from CST, USA; biotinylated *vicia villosa lectin* from Vector Laboratories.

### Coimmunoprecipitation (Co-IP) and immunoprecipitation (IP)

For Co-IP, cells transfected with indicated vectors were harvested and lysed. The whole cell lysates were immunoprecipitated with either anti-HA magnetic beads or anti-Flag magnetic beads (Bimake, China) and separated by SDS-PAGE followed by immunoblotting with the indicated antibodies. To confirm targeted proteins O-GalNAcylation, IP was performed. Cells were lysed with 50 mM Tris-HCl pH 6.8, 100 mM DTT, 2% SDS, 10% glycerol at 95 °C for 15 min. The denaturated lysates (59) were centrifuged and the supernatant was diluted with HEPES buffer (1:14) followed by IP with anti-HA magnetic beads and VVL agarose beads. The immunoprecipitated products were separated by SDS-PAGE and immunoblotted with VVL or anti-HA antibody.

### Quantitative real-time PCR

Total cell RNA was isolated using TRIzol (Invitrogen, Carlsbad, CA, USA), and cDNA was synthesized using an RT-PCR kit (TaKaRa, Japan) according to the manufacturer’s instructions. The cDNA was amplified by real-time PCR using primer sets specific for indicated genes, with *GAPDH* as an internal control. The sequences of the upstream and downstream primers are shown in Table S1. All target gene transcripts were normalized to *GAPDH*, and the relative fold change in expression was calculated using the 2^-ΔΔCt^ method.

### Fluorescent staining and confocal microscopy

Cells on coverslips were fixed in 4% paraformaldehyde/PBS for 15 min at room temperature (RT) and rinsed three times with PBS. Cells were permeabilized in 0.1% Triton X-100/PBS for 20 min and then blocked with 5% goat serum for 1h at 37 °C. Cells were incubated with primary antibodies against ppGalNAc-T4, E-cadherin, N-cadherin diluted in goat serum overnight at 4 °C. Cells were washed with PBS/0.05% Tween20 and secondary antibody (goat anti-rabbit IgG, conjugated with FITC), were used to visualize targeted proteins for 30 min at RT, washed again with PBS/0.05% Tween20, and then incubated with DAPI for 10 min at RT. Finally, cells were mounted with antifade reagent (Beyotime, China).

### CRISPR/Cas9-mediated genome editing

To generate GALNT4-knockout cells using CRISPR/Cas9 system, two single-guide RNAs (sgRNAs) targeting human *GALNT4* gene exon were cloned into Cas9 Vector. The sgRNAs sequences are 5′- GAATCCGGATGGCGGTGAGG TGG -3′ and 5′- CTGTTAAAAACGCCAGCAGC AGG -3′. After the sequences of insert confirmed by DNA sequencing, CRISPR/Cas9 plasmids were transfected into MCF7 cells. Twenty-four hours after transfection, positive cells were selected by puromycin (5 ng/ml) for 2 days, and then a single cell was isolated by limited dilution in 96-well plate. Further, the isolated cell was cultivated, and the region including the target site was amplified by PCR using the following primer, forward primer: 5′- TCTGGGCCTGGCGGACGAC -3′, and reverse primer: 5′- CTGTGAATGGTACGGAGCAAAGT -3′. The PCR product was ligated into pMD-19T cloning vectors (Takara), and ten cloned vectors were purified and verified by DNA sequencing at Sangon Biotech. For COSMC knockout, the sgRNAs sequences are 5′- ATGCTAGGACACATTAGGATTGG -3′ and 5′- GATGCATGTGATGATGTATGGGG -3′; PCR primers are 5′- GTTGCAAAACAAACTTCTCCATA -3′ and 5′- CACGCTTTTCTACCACTTCTCAG -3′.

### Cell transwell assay

The cell transwell chamber inserts of 24-well plate (pore size 8.0um, diameter 6.5 mm, Corning, USA) were used to examine the migration and invasion abilities of cells. A total of 600ul/well of serum-containing medium (20% FBS) was added to the lower chamber. After transiently transfected for 24 h, cells were treated with 5 μM mitomycin-C for 2 h to inhibit proliferation and resuspended at a density of 2 × 10^4^ per well in serum-free medium and added into the upper chamber. After incubation at 37 °C and 5% CO_2_ for 12 h, cells in the upper chamber were washed with PBS. Cells were fixed with 4% paraformaldehyde for 30 min at room temperature and stained with 0.1% crystal violet for 30 min at room temperature. Cells in the upper chamber were washed with PBS and removed with cotton swab. Cells were captured by microscope and counted in a random fashion by Image-Pro Plus software. For the invasion assay, ECMatrix gel (BD, USA) was melted overnight at 4 °C and coated the upper chamber according to the manufacturer’s introduction. Cells were incubated for 36 h, and the similar procedures were performed as described above. The independent experiments were run three times.

### Wound healing assay

Cells were seeded into 12-well plates at a density of 2 × 10^5^ cells/well in serum-containing medium at 37 °C and 5% CO_2_ until cell confluence was more than 95%. Cell confluent monolayers were wounded with pipette tips, and the floating cells were softly removed with PBS. Cells were cultured at 37 °C without 5% CO_2_ in fresh medium for 24 h and imaged using microscope. Images were analyzed to determine the percentage of the wound area coverage. The independent experiments were run three times.

### O-GalNAcylation reaction *in vitro* and HPLC assay

A total of 4 μg pFLAG-CMV-3-T2 and -T4 (Presented by Professor Zhang Yan of Shanghai Jiaotong University) was administered to 293T cells with 10 μl Lipo2000 (Invitrogen, UK) according to the manufacturer's instructions. The cells were incubated with DMEM medium after 6 h, and the culture supernatant was collected after 48 h. The culture supernatants were incubated for 12 h at 4 °C with 50 μl Anti-FLAG M2 beads (Sigma, USA). The beads were washed three times with 1 ml TBS (50 mM Tris-HCl, 150 mM NaCl, pH 7.4) and then eluted with 15 μg 3 × Flag (Sigma, USA) in 50 μl TBS at 4 °C, and ppGalNAc-T2 and -T4 enzymes were in the supernatant. O-GalNAcylation enzymatic reaction *in vitro* and HPLC assay were also performed as previously described (52). The enzymatic reaction system (20 μl) consisted of 50 ng/μl ppGalNAc-T2 or ppGalNAc-T4 enzyme, 250 μM UDP-GalNAc (Sigma, USA), 25 μM FAM-labeled peptide (ChinaPeptides Co,Ltd), 0.2% Triton 100, 5 mM MnCl_2_, 25 mM Tris-HCl (pH 7.4), and Merck H_2_O. The enzymatic reaction was carried out at 37 °C for 9 h, and the sample was boiled at 95 °C for 5 min to terminate the reaction. The acceptor peptides were derived from the sequence of human TβR Ⅰ and Ⅱ, respectively. *In vitro* O-glycosylation products were evaluated by RP-HPLC (Shimadzu, Japan) on a C18 analytical column (5C18-AR-II, 4.6 × 250 mm, Cosmosil, Japan). Peptides were loaded onto a C18column at a flow rate of 1 ml/min and were detected at 495 nm with a fluorescence detector. Mobile phase A consisted of 0.1% formic acid (Sigma, USA), and mobile phase B was 0.1% formic acid in ACN (Sigma, USA). A linear gradient of 20–28% B in 16 min, 28–80% B in 4 min, 80% B for 3 min, and 80–20% B in 3 min was employed throughout this study. The addition of GalNAc to the peptide may induce conformational changes of the peptide that increase the hydrophilicity of the peptide. Therefore, the retention time of the peptide bound to GalNAc was earlier than that of the unmodified peptide through the reversed-phase C18 column. *K*_*m*_ values for acceptor substrates were calculated using peptides with concentrations from 0.001 to 0.1 mM in the presence of 0.5–1.0 mM uUDP-GalNAc. The Michaelis–Menten equation was applied to the initial rate data using a nonlinear square regression program.

### Mass spectrometry

FAM-labeled peptide was subjected to an enzymatic reaction with ppGalNAc-T4 enzyme *in vitro*. The reaction mixture was desalted by buffer exchange into 40 mM ammonium bicarbonate using a spin column (10 kDa cutoff, Millipore, USA). The sample was reduced with 100 mM DTT (Solarbio, China) for 5 min at 100 C and then carboxyamidomethylated with 100 mM iodoacetamide (Solarbio, China) dissolved in UA solution (8 M urea in 0.1 M TrisHCl, pH 8.5) in the dark for 30 min. After centrifugation at 14,000g for 15 min, the filtrate was discarded, and the protein was adsorbed on the membrane of the spin column. Sequencing-grade trypsin [1:50 (w/w)] (Promega, USA) with 40 mM ammonium bicarbonate was added to the spin column. The protein was digested at 37 °C overnight for MS.

For LC-ESI-MS, peptides and glycopeptides were analyzed on a capillary LC-ESI-MS system comprising an Aquasil C-18 precolumn (Thermo Scientific, USA, 30 mm × 0.32 mm, 5 mm), a BioBasic C18 analytical column (Thermo Scientific, USA, 150 mm × 0.18 mm, 5 mm), a Waters CapLC, a Rheodyne 10-port valve, and a Waters QTOF Ultima with a standard ESI-source. Phase A consisted of 65 mM ammonium formate at pH 3.0, and phase B was 80% ACN in phase A. The precolumn was equilibrated and loaded in the absence of ACN. Thereafter, a gradient from 6.3 to 62.5% phase B was developed over 45 min. The positive ions in the range from m/z 500 to 2000 were measured. The capillary voltage was 2.25 kV, the cone voltage was 35 V, the source temperature was 100 °C, and the desolvation temperature was 120 °C. The data were evaluated using MassLynx 4.0 software. A potential variable modification of 203.08 Da on serines/threonines was considered during searches to identify potentially O-GalNAc-modified residues.

### Structure modeling, molecular dynamics (MD) simulation, and conformational landscape analysis

For the N-terminal residues missing from Met1 to Asn42 in all of the TβR Ⅱ crystal structures in Protein Data Bank (PDB), we have to build part of the N-terminal structure to contain Ser31, which we are focused on. Eight structures (PDB ID: 1KTZ, 1M9Z, 1PLO, 2PJ, 3KFD, 4P7U, 4XJJ, and 5E8V) were used as the templates for Modeller 9.12 to build totally 2000 TβR Ⅱ structures from Gln29 to Phe121. Three modeling results with the top-rank DOPE and Molpdf scores were selected to check the stereochemical quality by the PROCHECK program. The best modeling result with the highest favored/allowed rate in Ramachandran plot was chose to run a short (10 ns) molecular dynamics simulation with TβR Ⅰ as a heterodimer to relax the structure.

All of the MD simulations were performed by Gromacs 2019.2 with Amber14 force field. The modified structure with O-GalNAcylation (GalNAcαSer31) was built by the Glycprotein Builder on GLYCAM-Web server. The protocol of the MD simulation was as follows: 1) The complex structures, both wild-type and glycosylation modified systems, were solvated in a cubic TIP3P water box with 1 nm distance from the edge and relaxed using 2000 steps of steep descent minimization followed by 5000 steps of conjugate gradient minimization; 2) the complexes were then equilibrated under standard NVT and NPT conditions for 1 ns, respectively; 3) after the equilibration run, a 100 ns simulation at constant pressure with a target temperature of 300 K and pressure of 1 atm was conducted for each system. Particle mesh Ewald (PME) method implemented in Gromacs 2019.2 was used to treat the long-range electrostatic interactions in the production phase. The LINCS algorithm was employed to restrain the hydrogen positions at their equilibrium distances; 4) both energies and coordinates were saved every 10 ps for the postproduction analysis.

The conformational landscapes were obtained by integrating the deposited bias during the metadynamics algorithm implemented in PLUMED plug-in of VMD program. For convenience, they were shown as a function of two distances at a time (CV1, distance-1; CV2, distance-2).

## Data Availability

All data are contained within the article.

## Conflict of interest

The authors declare that they have no conflicts of interest with the contents of this article.
