# Supplementary material for: ppGalNAc-T4-catalyzed O-Glycosylation of TGF-β type Ⅱ receptor regulates breast cancer cells metastasis potential
Source: J Biol Chem. 2020 Dec 3;296:100119. doi: 10.1074/jbc.RA120.016345 (PMC7948473; doi:10.1074/jbc.RA120.016345)
Supplement: Figures and Table [file mmc7.docx]

**Supporting information**

**ppGalNAc-T4-catalyzed O-Glycosylation of TGF-β type Ⅱ receptor**

**regulates breast cancer cells metastasis**

Qiong Wu^1^, Cheng Zhang^1^, Keren Zhang^2^, Qiushi Chen^2^, Sijin Wu^3,4^, Huang Huang^1^, Tianmiao Huang^1^, Nana Zhang^1^, Xue Wang^1^, Wenli Li^1^, Yubo Liu^*,^ ^1^, Jianing Zhang^*, 1^

**A list of the material included**

Table S1

Figure S1-S8

Movie 1-6

**Table. S1 qPCR primers**

| **Gene** | **Forward primer 5’—3’** | **Reverse primer5’—3’** |
| --- | --- | --- |
| ***GALNT4***  ***GADPH***  ***ZO1***  ***TCF8***  ***CDH2***  ***CDH1***  ***SNAI1***  ***GALNT1***  ***GALNT2***  ***GALNT3***  ***GALNT6***  ***GALNT7***  ***GALNT8***  ***GALNT11***  ***GALNT12***  ***GALNT14***  ***GALNT16*** | GTCAAGAAGGCTCTCAGACCTC  TCCAAAATCAAGTGGGGCGA  AGGAGGTAGAACGAGGCATCATCC  CCAGGCTGAGGTATTCCTTGTTGC  GTCAACATCACTCTCACCGA  AATACATCTCCCTTCACAGCA  GGCTCCTTCGTCCTTCTCCTCTAC  TGGATATGTTCCTGCTGCTTTA  GGTGGCGGAGGACAGGACTC  GCGTTGGTCAGCCTCTATGTCTG  GAC AAG ACA GTG GTG GTG AG  TGCTGGAGGAGATTCCCAGAA  ACACTGAAGAGGGCGAAAGA  CCAGGACACCATGACACACAACTC  GCCCAGGACATCTGTTATCATA  TGTAAACAGCTCATCAAGTTGC  TACTACTTATGGCAGGACAACC | GTTCATCCTCGTTGAGCTGGAG  AAATGAGCCCCAGCCTTCTC  TCTCCAGAAGTCAGCACGGTCTC  CACTTGCTCACTACTCTCGCCTAC  ATCCACAGCTCTTATTCTTCCA  TAGCAAAGCAAGAATTCCTCC  CCAGGCTGAGGTATTCCTTGTTGC  GTACTGGCTCTAGAACATCTCC  CTCTGCTCAGGCGTCATGTAATCC  AACGAGACCTTGAGCAGCATGAAG  GAA GGT CAG GCT CCA GTC A  GCACAGGATCATGGTAGGTGAA  AAATGAGCCCCAGCCTTCTC  TTCCACACGCTCACTGATATTGCC  GCCCAGGACATCTGTTATCATA  TGAAGGTGTCCAGGTTAATGAT  CTTCTCATCAAAGCCTTTCGAG |

**Figure S1**

**A**


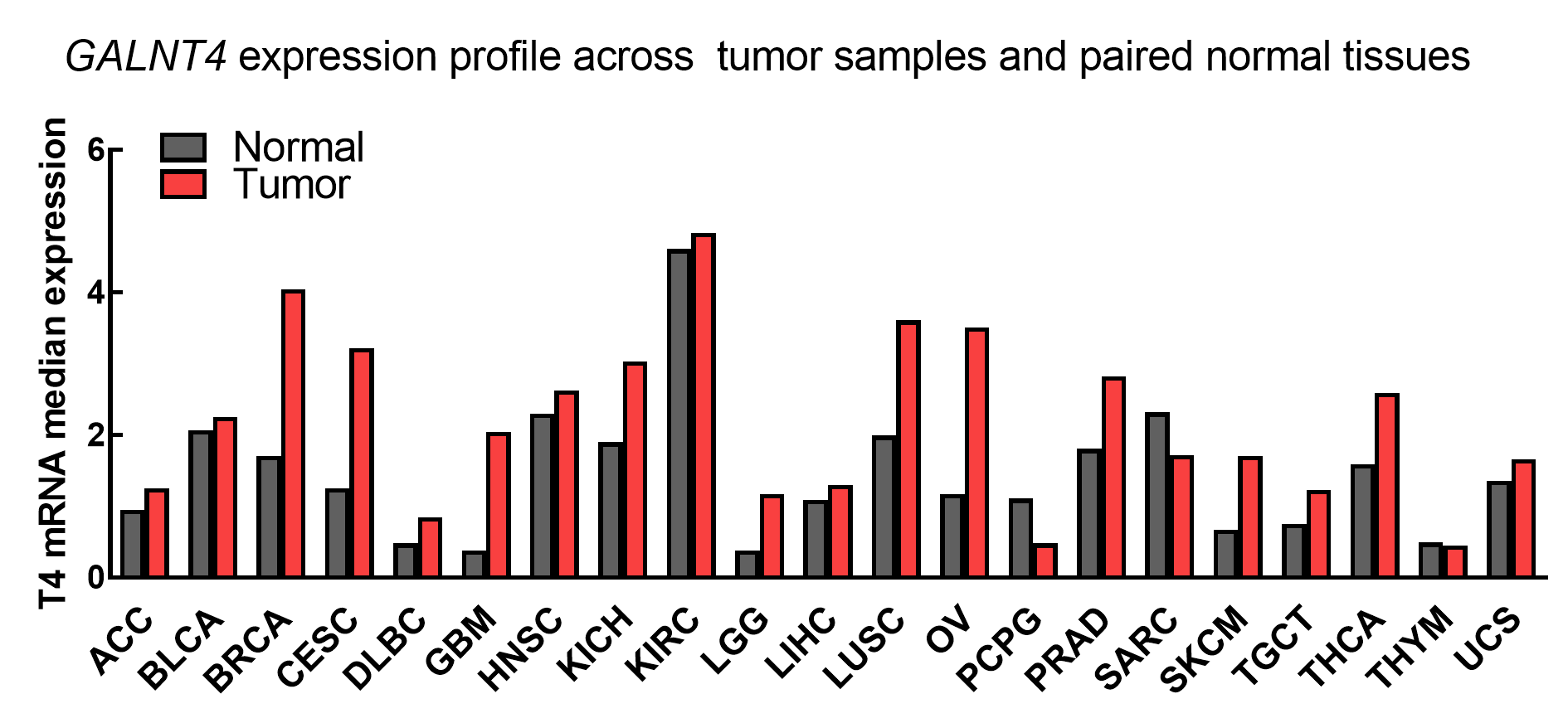


**B**


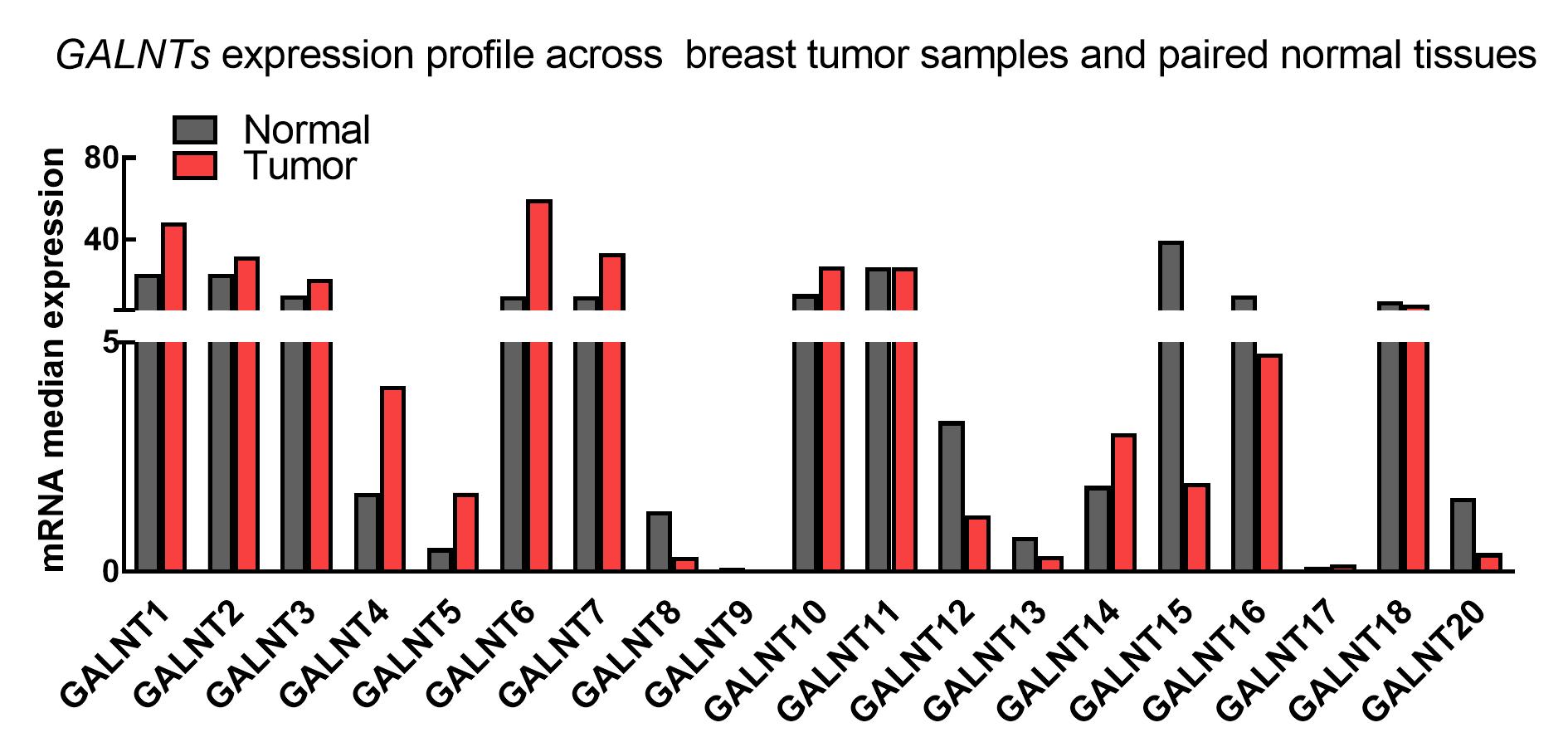


**Fig. S1** (A) *GALNT4* expression profile across tumor samples and paired normal tissues. *GALNT4* mRNA expression profile across multiple tumor samples and paired normal tissues were checked using online datasets (GEPIA). Among these multiple cancer and paired tissues, *GALNT4* showed differential expression level in breast cancer and paired normal tissues. (B) *GALNTs* expression profile across breast tumor sample and paired normal tissues. (data from GEPIA). Other *GALNTs* expression profile in breast cancer and paired normal tissues were checked. Besides *GALNT4*, *GALNT5*, *GALNT8*, *GALNT12*, *GALNT15* and *GALNT20* also showed differential expression level between breast tumors and paired normal tissues. This data suggested that not only ppGalNAc-T4 but also other several isoforms might participate in breast cancer progression. Further research is needed to reveal the function of these ppGalNAc-Ts in breast cancer progression.

**Figure S2**

**A**


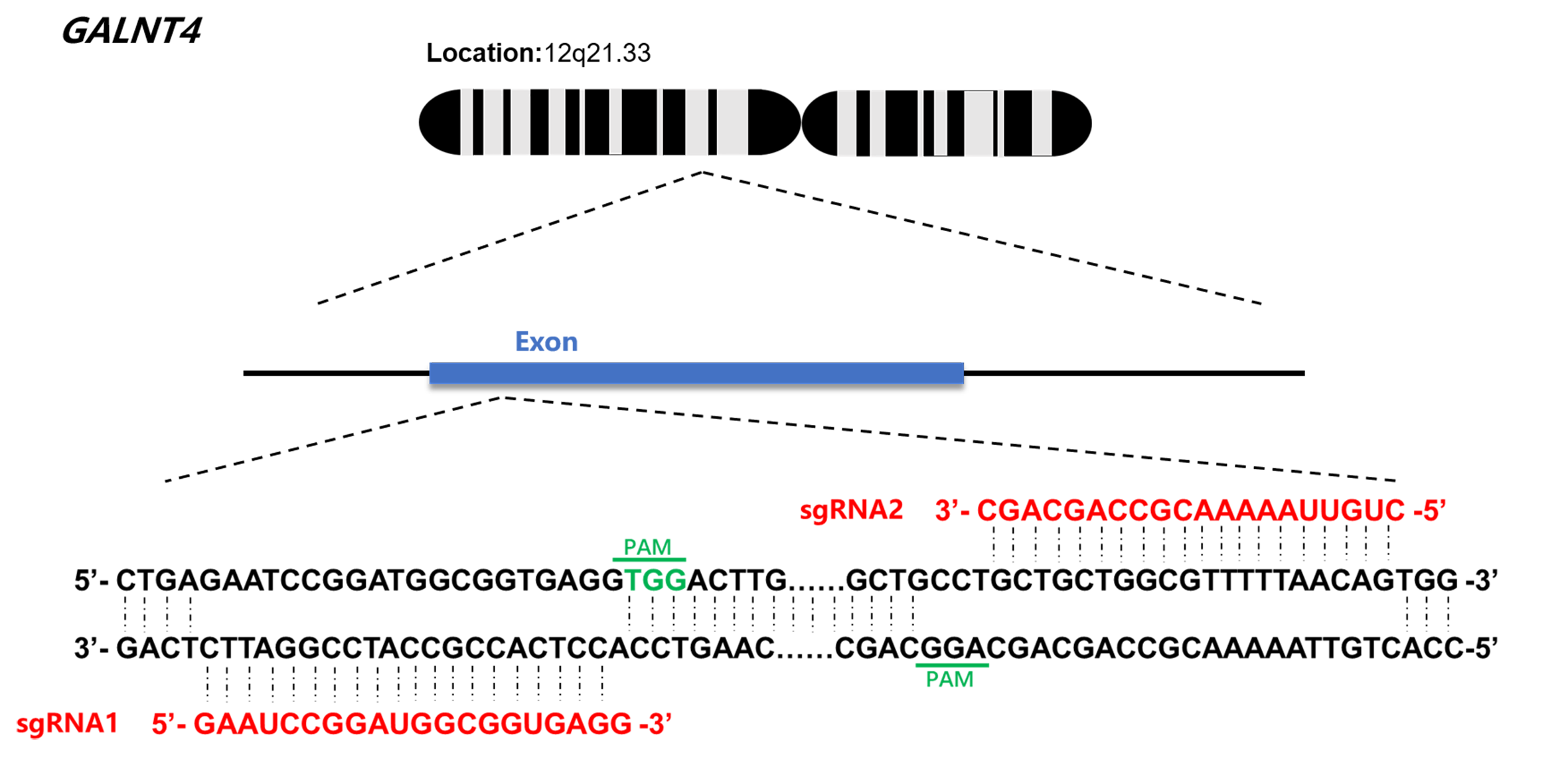


**B**


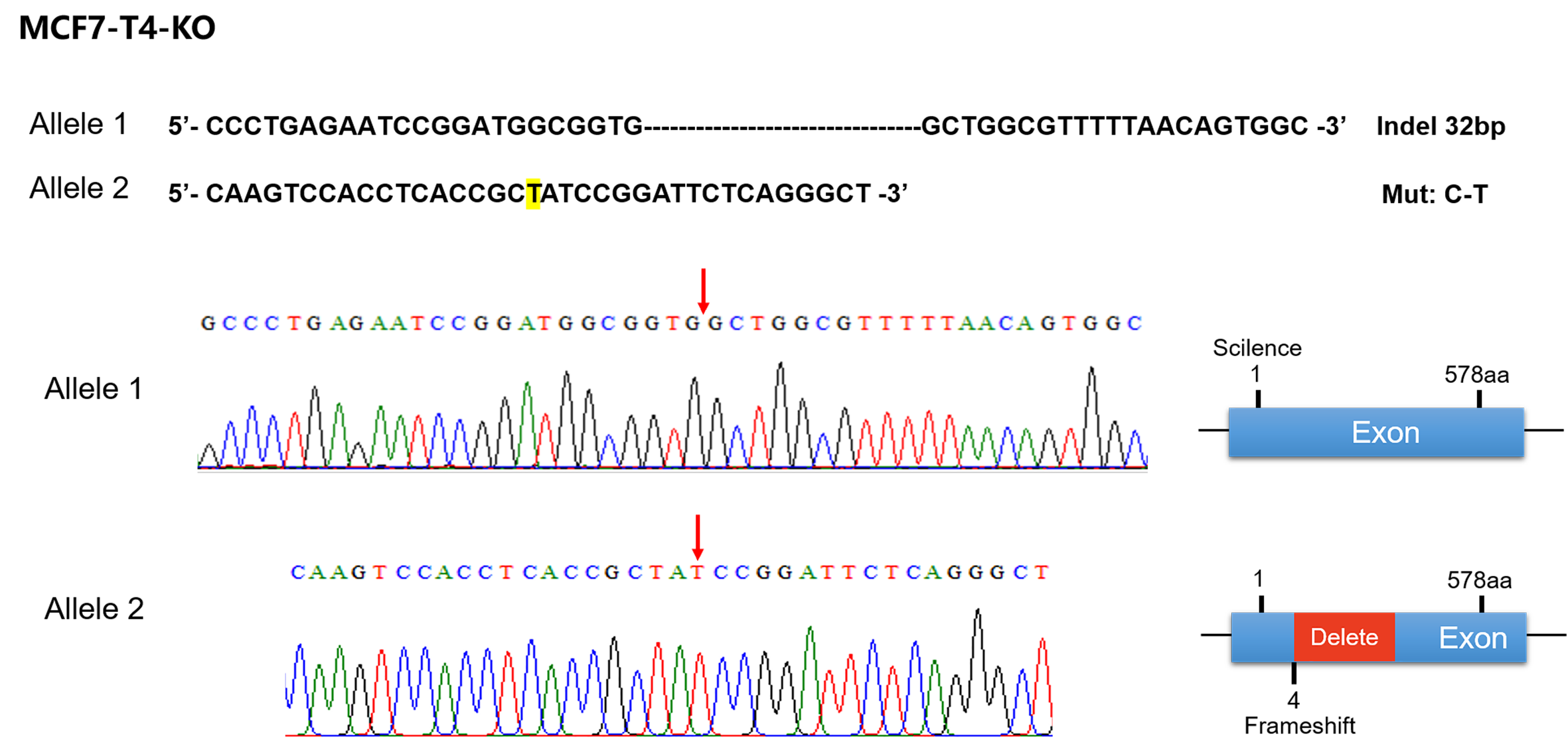


**Fig. S2 Generation of ppGalNAc-T4 knockout cell using CRISPR-Cas9 genome editing.** (A) Schematic diagram of the base pairs between single-guide RNAs targeting human *GALNT4* gene. (B) The sequences of the mutated alleles in *GALNT4* knockout cell.

**Figure S3**


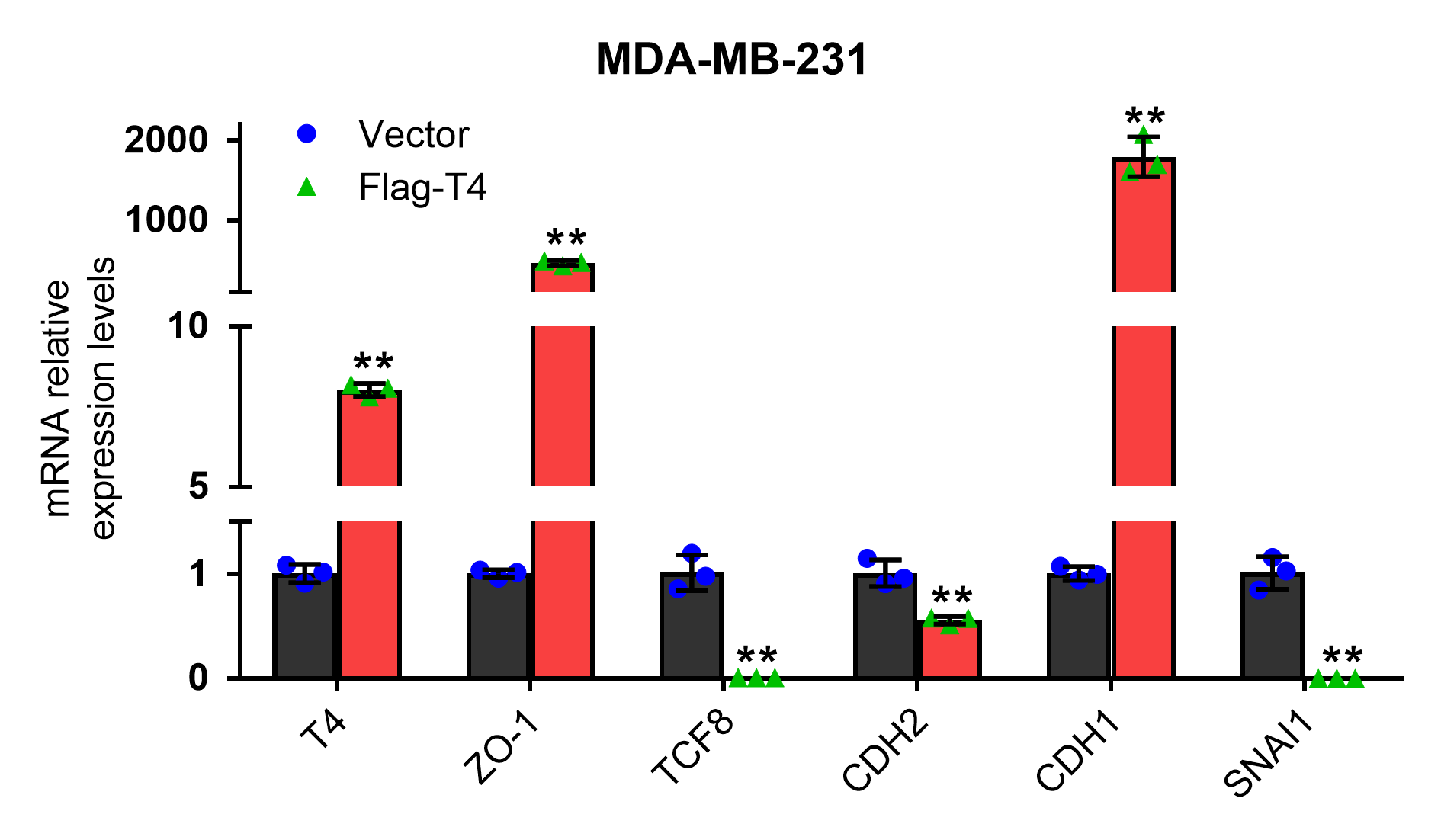


**A**

**B**


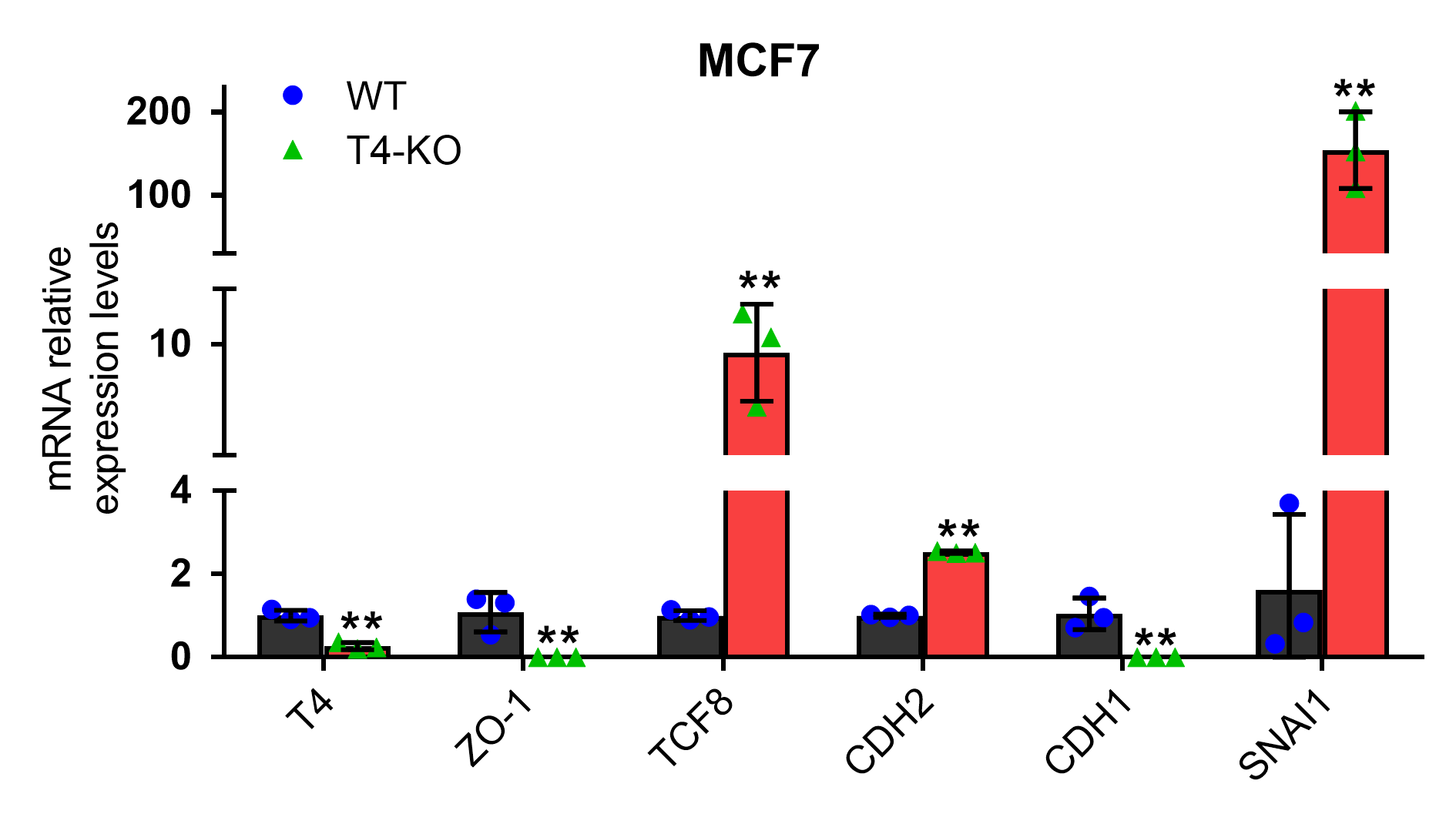


**Fig. S3** *GALNT4*, *ZO-1*, *TCF8*, *CDH2* (gene name of N-cadherin), *CDH1* (gene name of E-cadherin) and *SNAI1* mRNA expression levels were examined by qPCR after ppGalNAc-T4 was regulated in MDA-MB-231 (A) and MCF7 cells (B).

**Figure S4**

**A**


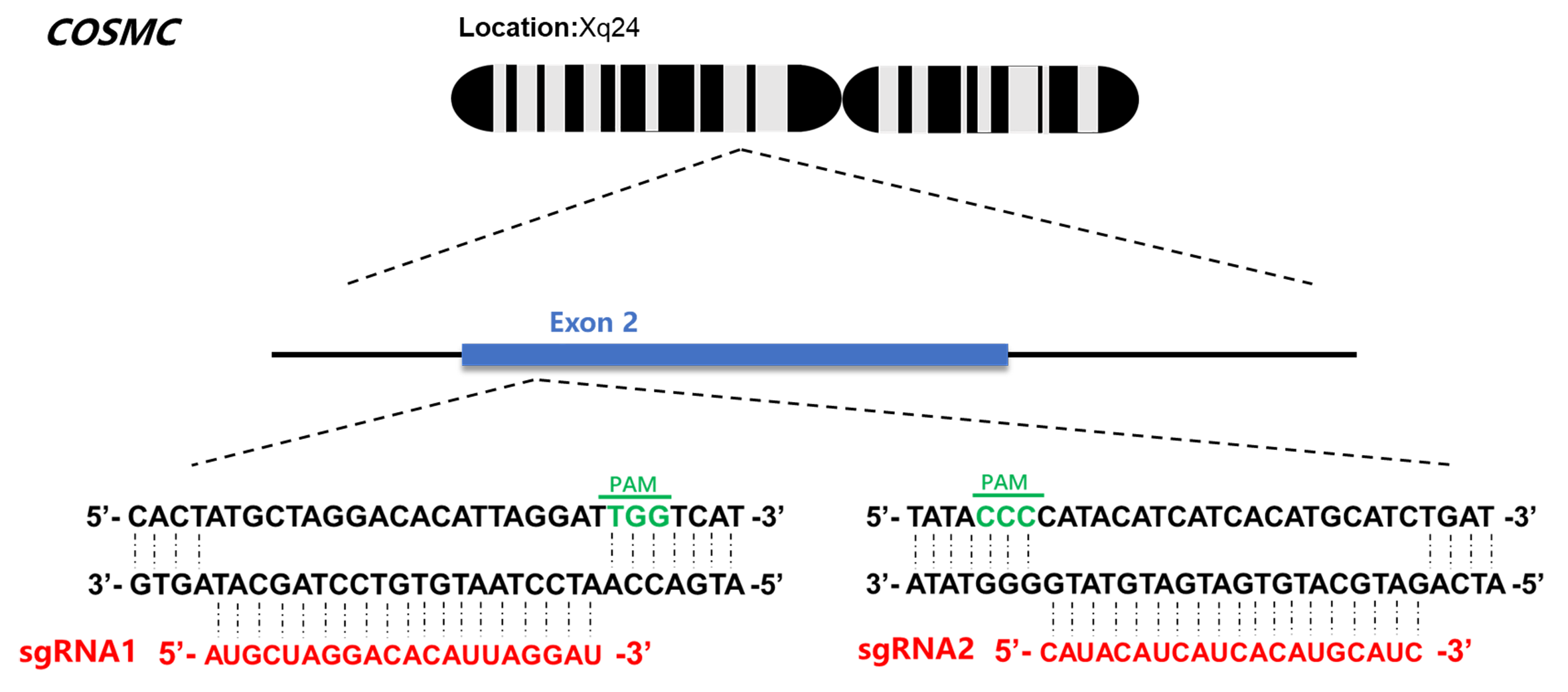


**B**


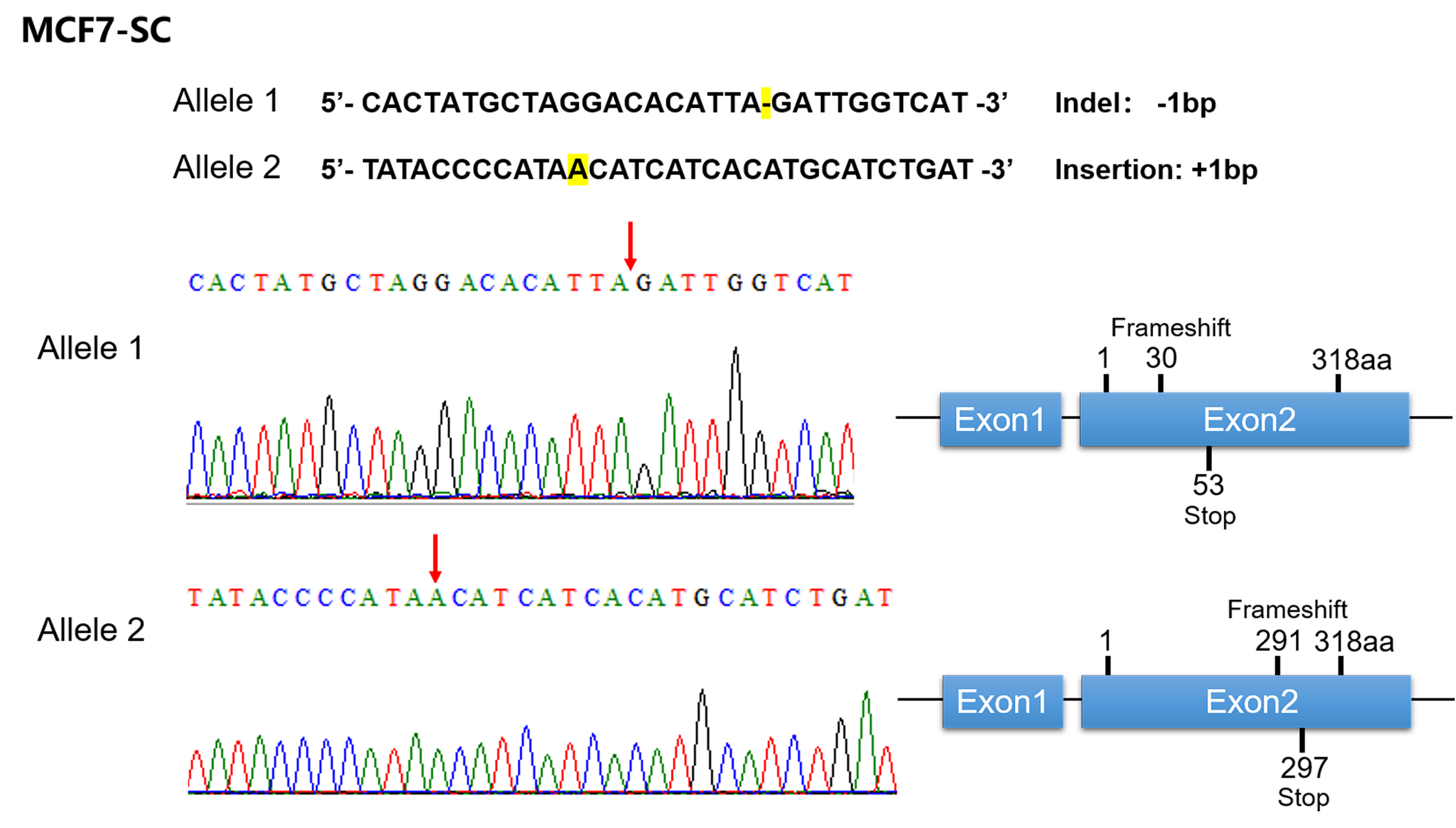


**C**


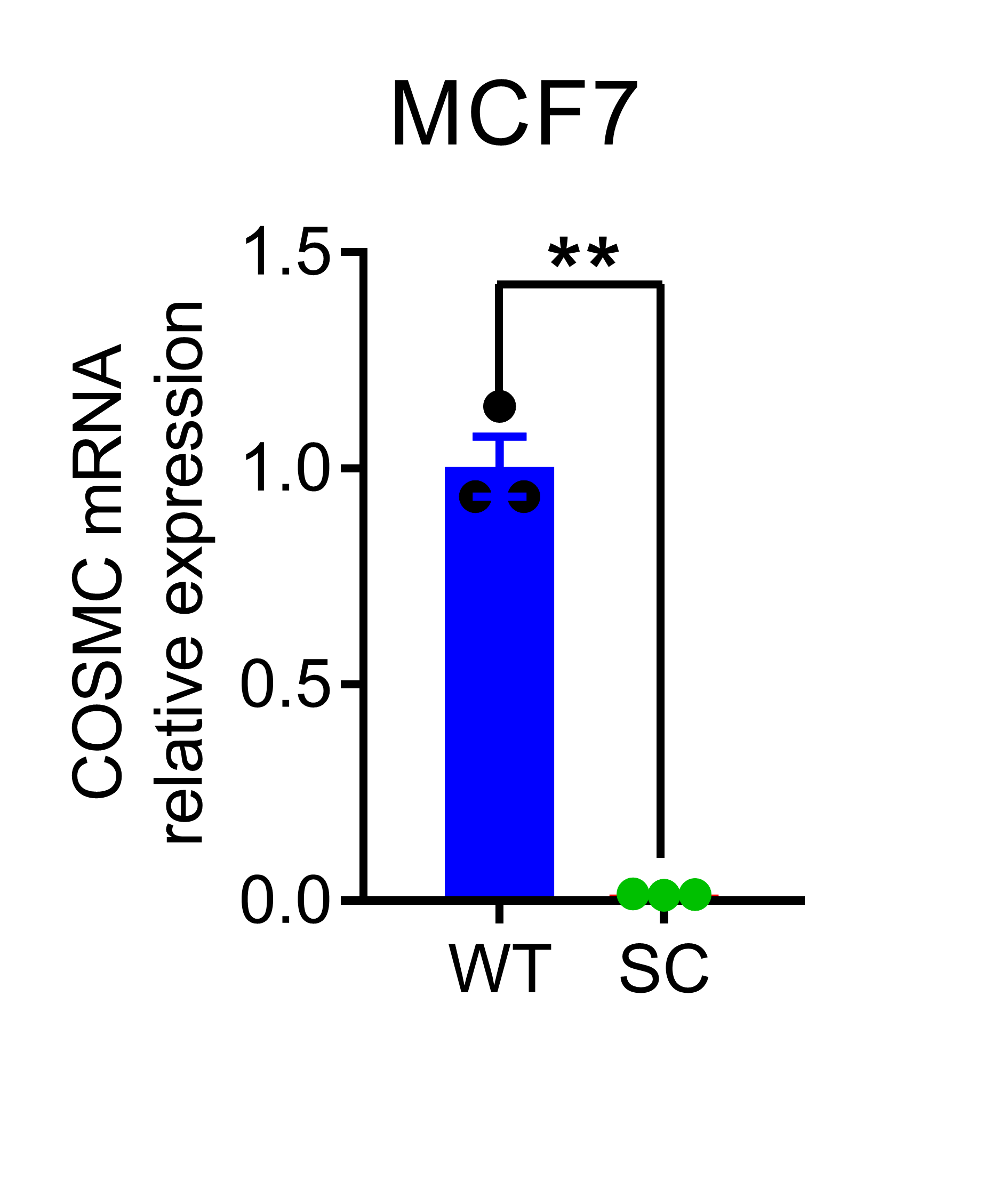


**Fig. S4 Generation of COSMC knockout cell using CRISPR-Cas9 genome editing.** (A) Schematic diagram of the base pairs between single-guide RNAs targeting human *COSMC* gene. (B) The sequences of the mutated alleles in *COSMC* knockout cell. (C) Validation of *COSMC* gene knockout by qPCR.

**Figure S5**

**A**


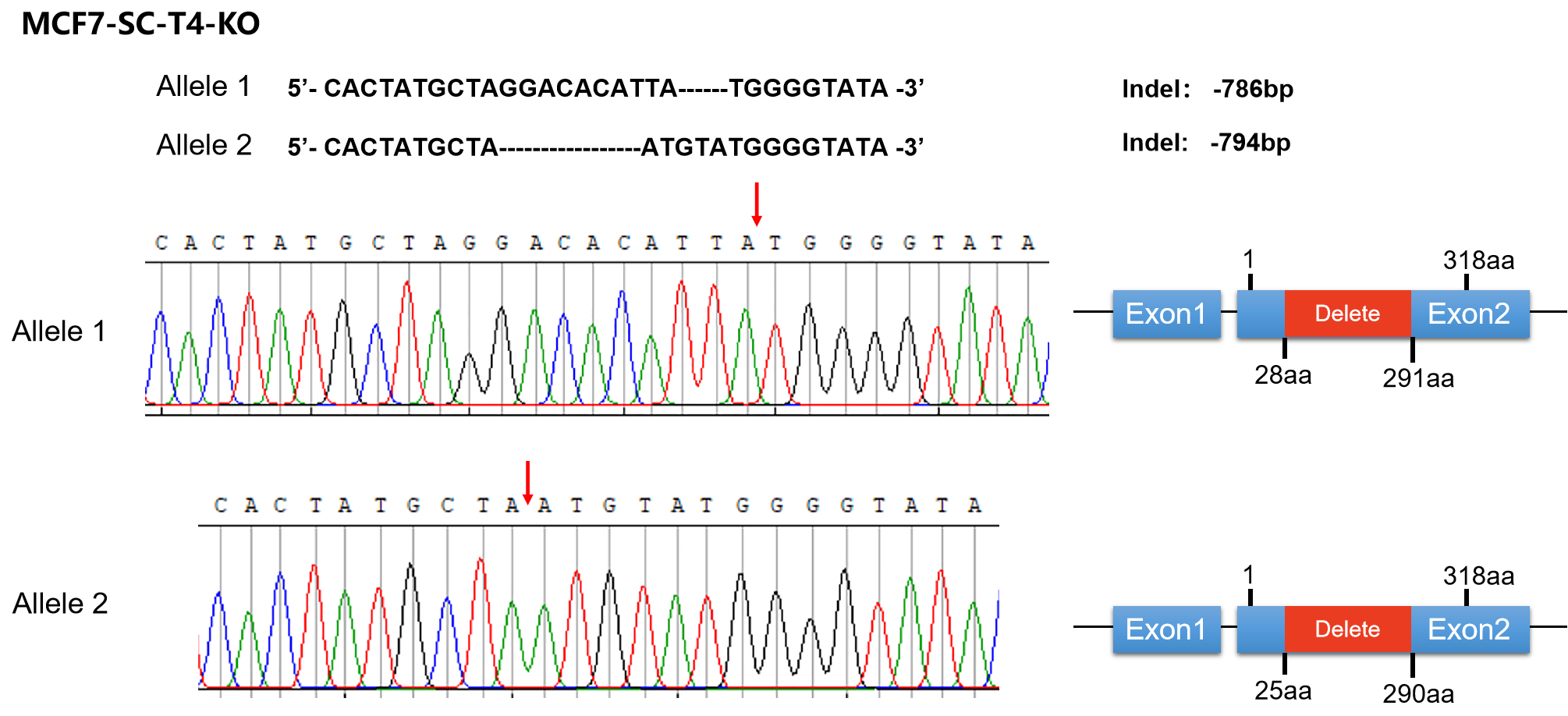


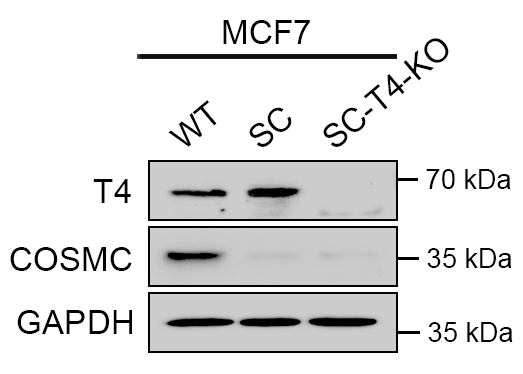

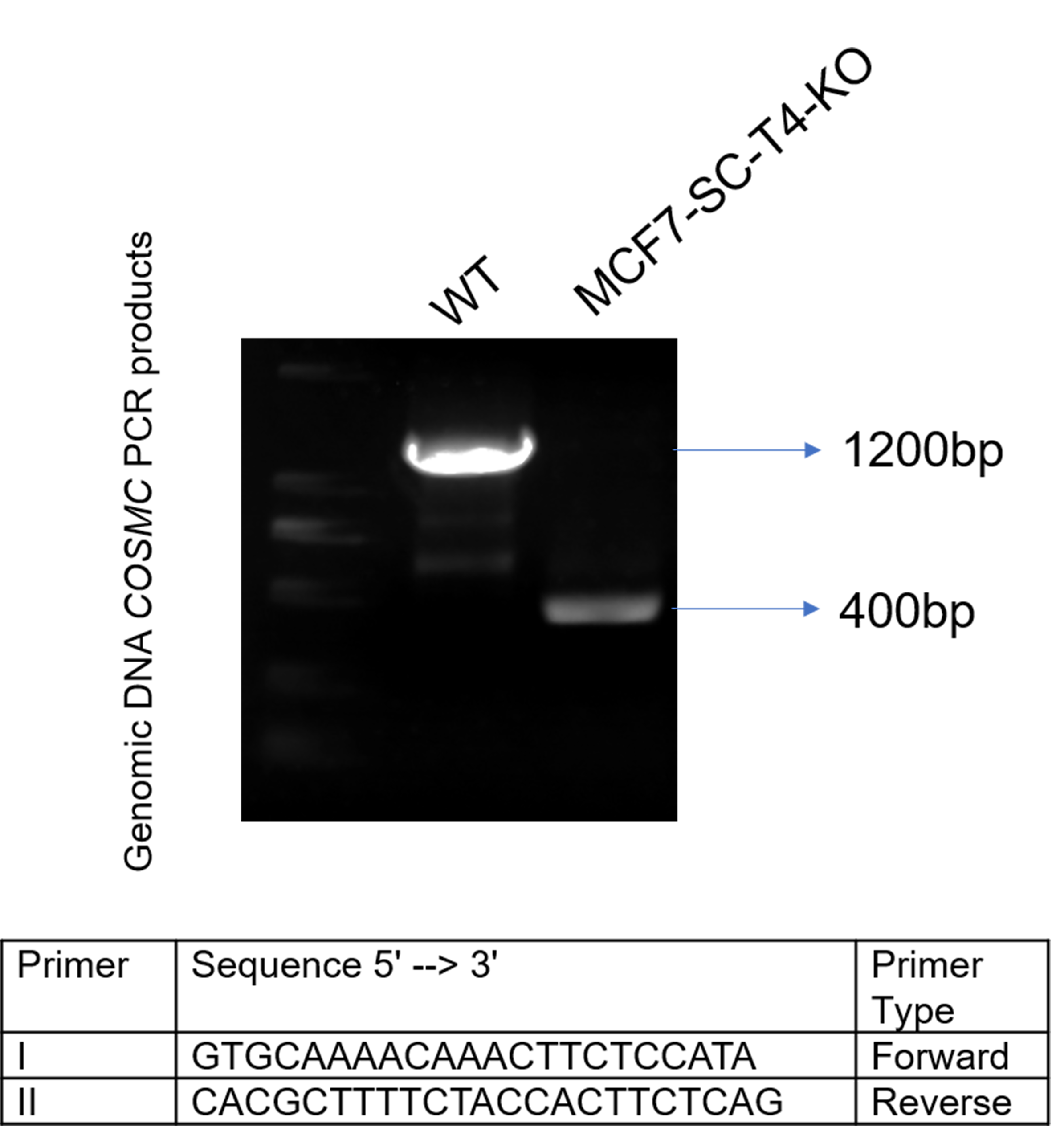


**C**

**B**

**Fig. S5 Generation of *GALNT4/COSMC* double gene knockout cell using CRISPR-Cas9 genome editing.** (A) The sequences of the *COSMC* gene mutated alleles in *GALNT4/COSMC double* gene knockout cell. (B) Validation of genome *COSMC* gene knockout by PCR. (C) Validation of ppGalNAc-T4 and COSMC knockout by western blot.

**Figure S6**

**B**

**A**


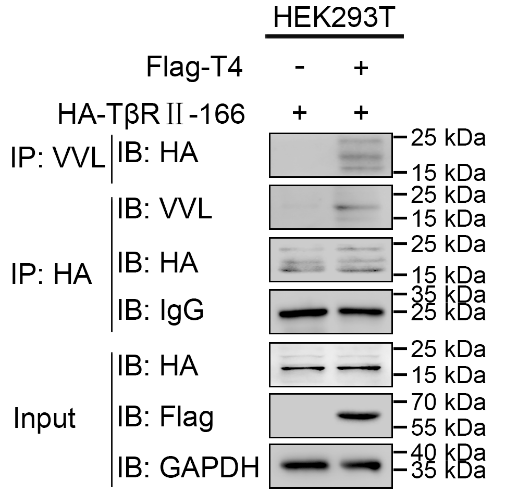

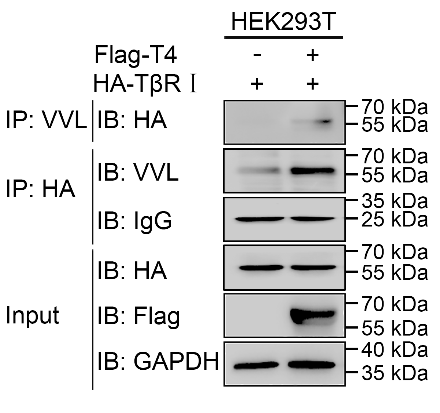

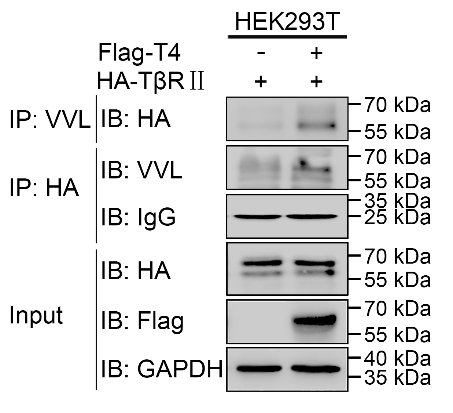


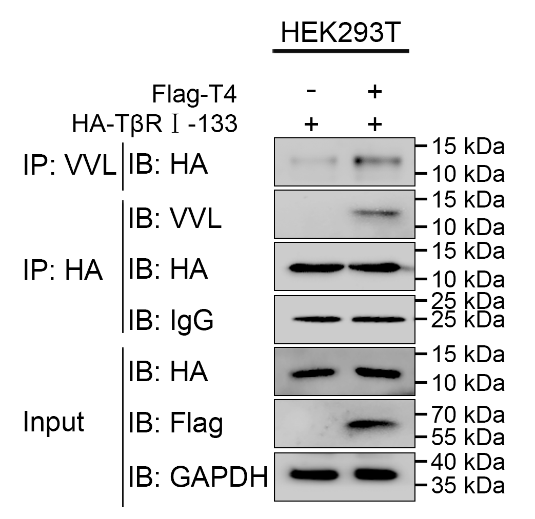


**D**

**C**

**Fig. S6 ppGalNAc-T4 catalyzed O-GalNAcylation occurs of TGF-β receptors in HEK-239T cell line.** (A) HA-TβR Ⅰ or (B) HA-TβR Ⅱ and Flag-T4 were transfected into HEK-293T cell. The cell lysates were immunoprecipitated using agarose bound VVL and anti-HA magnetic beads. IP: anti-VVL, IB: anti-HA; IP: anti-HA, IB: anti-VVL. The inputs and immunoprecipitates were examined by western blot using corresponding antibodies. (C) Extracellular domain of TβR Ⅰ, HA-TβR Ⅰ-133 or (D) extracellular domain of TβR Ⅱ, HA-TβR Ⅱ-166 as well as Flag-T4 were transfected into HEK-293T cell. The cell lysates were treated as in Fig. 4G and 4B. IP: anti-VVL, IB: anti-HA; IP: anti-HA, IB: anti-VVL. The inputs and immunoprecipitates were examined by western blot using corresponding antibodies.

**Figure S7**


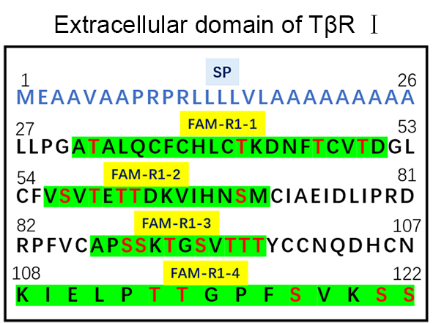

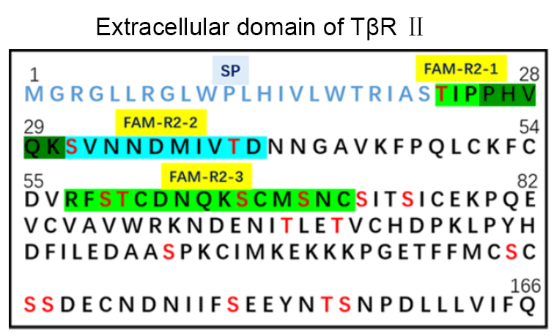


**B**

**A**


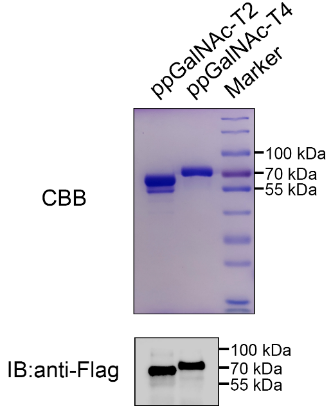


**D**

**C**


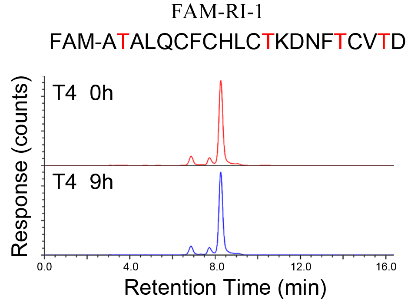


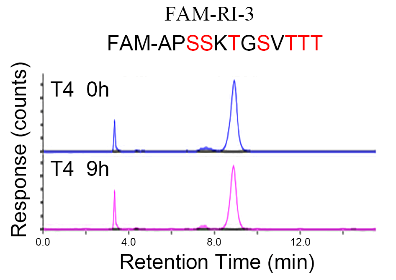

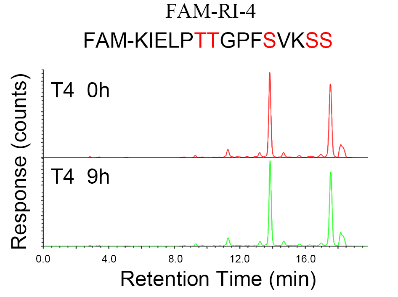

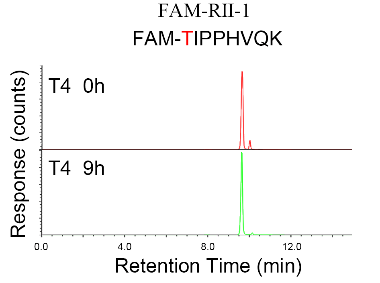


**F**


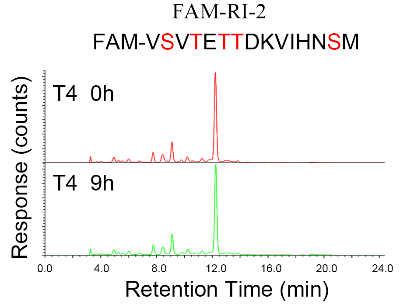


**E**

**H**

**G**

**H**

**G**

**G**

**G**


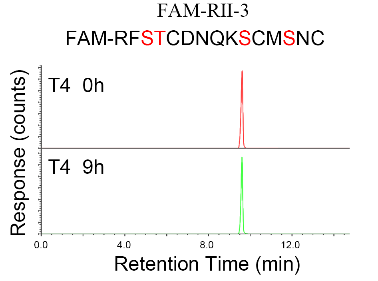


**I**

**Fig. S7** Detecting O-glycosylation of peptides based on part of TGF-β receptor sequences *in vitro* by HPLC assay. (A) and (B) Position and sequence of multiple candidate peptides of TβR Ⅰ and R Ⅱ extracellular domain containing putative O-GalNAcylation sites for probing the modification. (B) Recombination of ppGalNAc-T2-Flag and -T4-Flag enzymes were expressed in HEK-293T cell and purified with anti-Flag magnetic beads. Purified products were confirmed by coomassie brilliant blue (CBB) and immunoblotting with anti-Flag antibody. (D), (E), (F), (G), (H) and (I) Products derived from ppGalNAc-T4 reaction of TβR Ⅰ and R Ⅱ peptides with possible O-GalNAcylation sites were analyzed by HPLC.

**Figure S8**

**A**


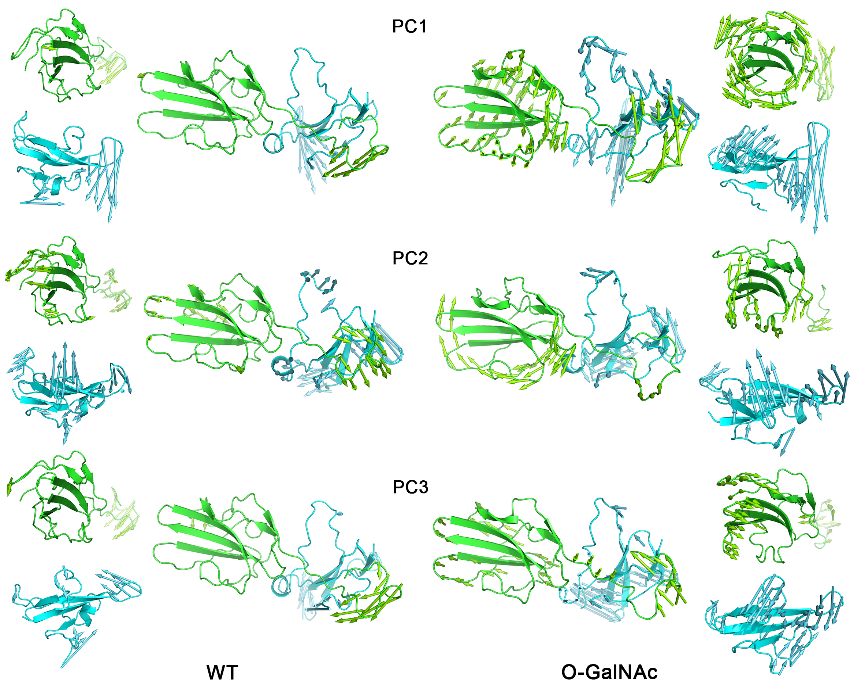


**B**


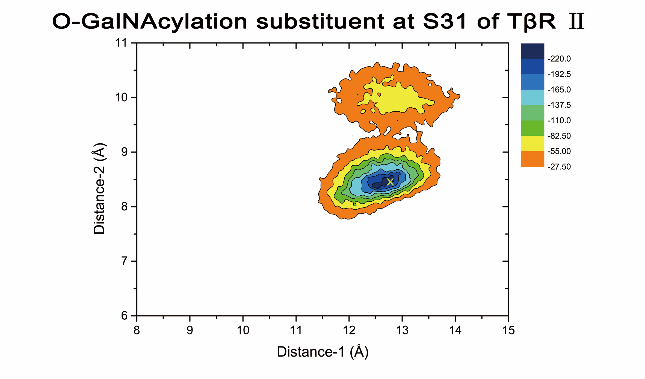

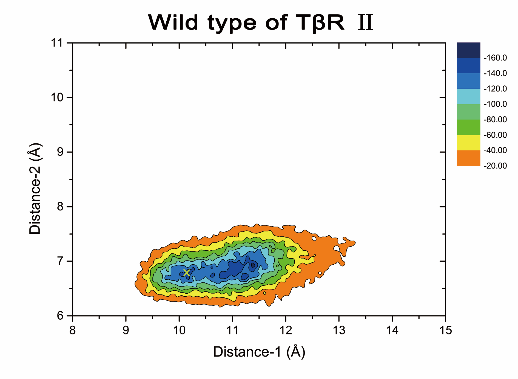


**C**


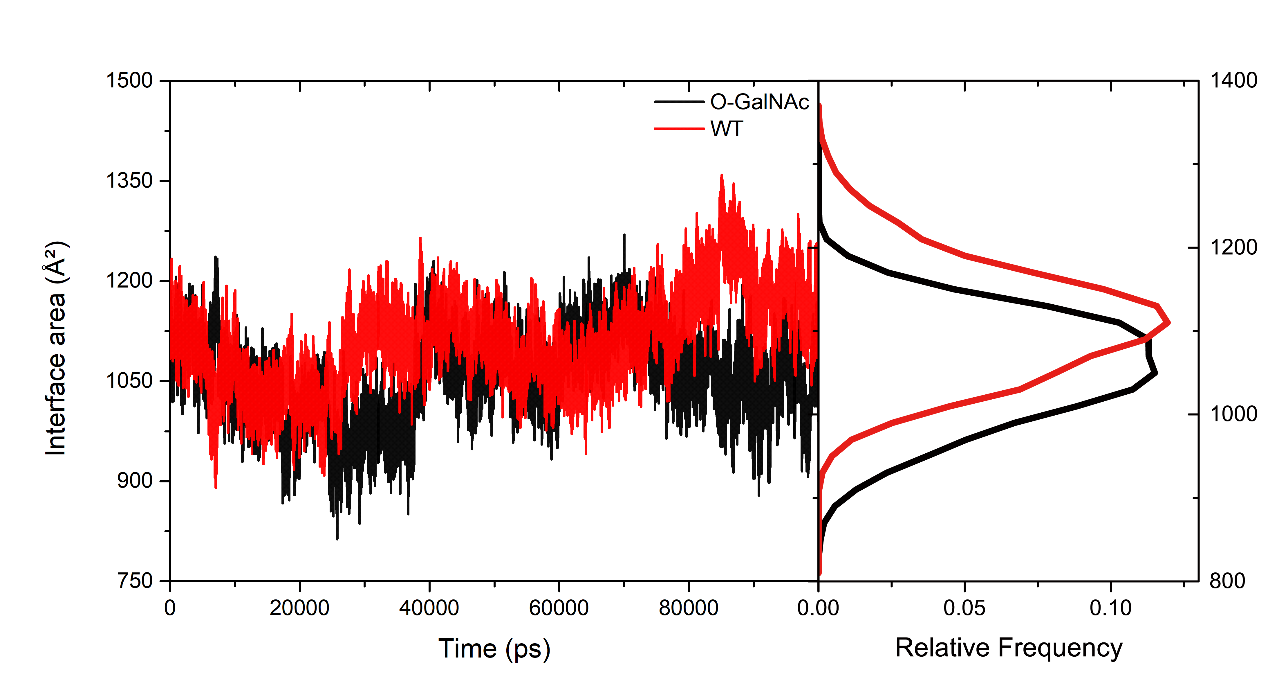


**Fig. S8 Structural modeling, molecular dynamics simulation and conformation landscape analysis of TβR Ⅱ.** (A) The conformational changes of PC1, PC2 and PC3. TβR Ⅱ is shown as green cartoon, and TβR Ⅰ is shown as a cyan cartoon. For each figure, one front view and two side views of TβR Ⅰ and TβR Ⅱ, respectively, are shown. The arrows show the motion between two extreme conformations of each PC with green and cyan for TβR Ⅱ and TβR Ⅰ, respectively. (B) The interface areas between TβR Ⅰ and TβR Ⅱ. Left: the interface areas as a function of time in two different systems derived from the MD simulation run. The black line corresponds to the O-GalNAcylated (GalNAcα) substituent system, and the red line corresponds to the wild-type system. Right: the relative frequency of the interface areas in these two systems, which indicates that the interface area of the O-GalNAcylated system is significantly smaller than that of the wild-type system. (C) The interface areas of TβR Ⅰ and TβR Ⅱ. Left: the interface areas as a function of time in two different systems derived from the MD simulation run. The black line corresponds to O-GalNAcylated (GalNAcα) TβR Ⅱ, and the red line corresponds to the wild-type system. Right: the relative frequency of the interface areas in these two systems, which indicates that the interface area of the TβR Ⅱ system is significantly smaller than that of the wild-type system.

**Figure S9**


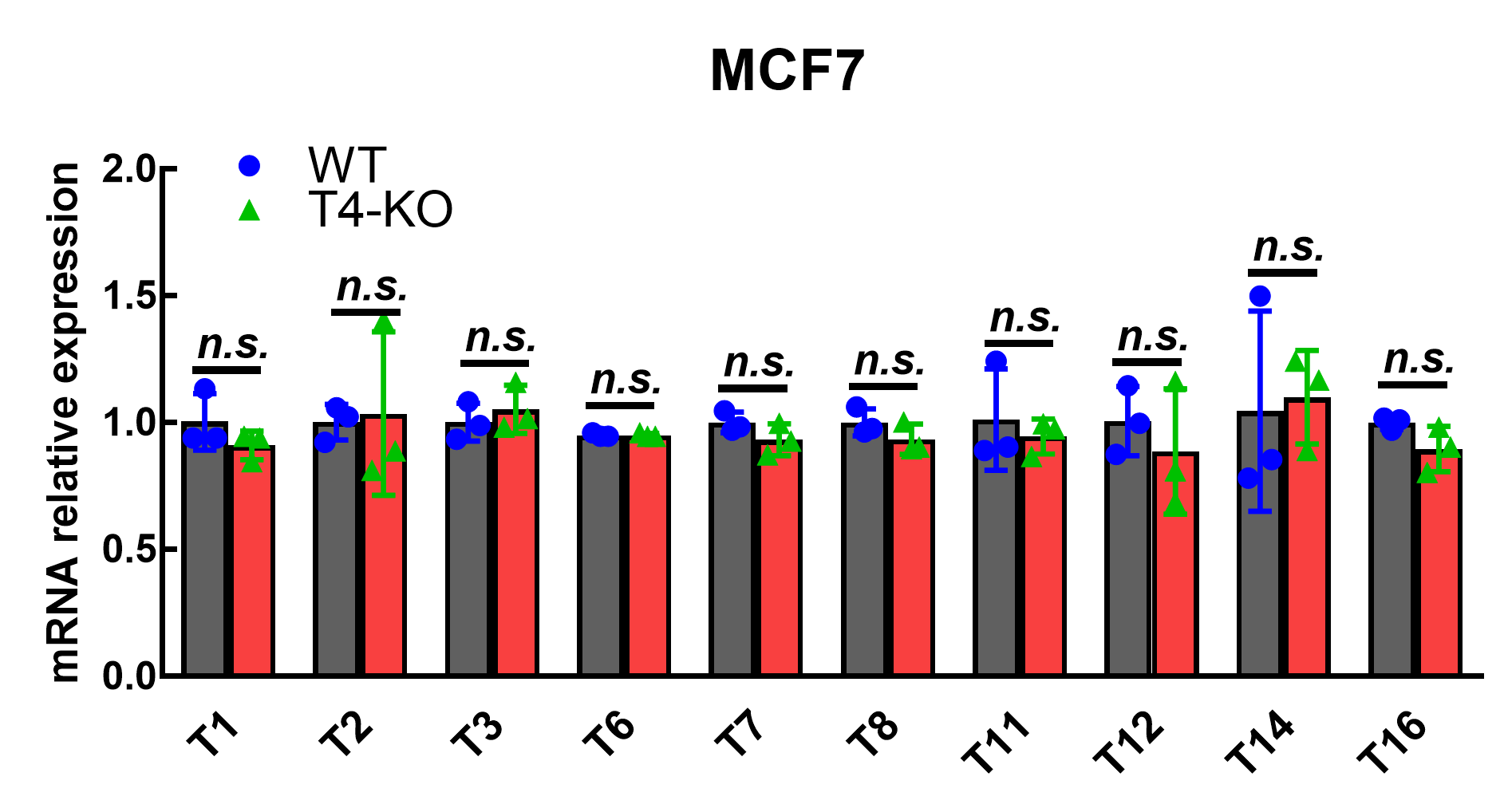


**Fig. S9** Ten ppGalNAc-Ts mRNA relative expression levels by qPCR after ppGalNAc-T4 was knockout in MCF7 cell. The mRNA expression levels of T2, T3, T6, T11, T14 and T16, which have been reported involved in TGF-β receptors or signaling were checked. T12, which is clustered in the same subgroup with T4 in family evolutionary tree was check. We also checked the expression levels T1, T7, T8. The sequences of primers were listed in supplementary Table. S1.

**Movie 1-6**

The motion of 3 principal component (PC1, PC2, PC3) conformational changes upon TβR Ⅰ and TβR Ⅱ binding. Movie 1-3, GalNAcα substituent at Ser31 of TβR Ⅱ; Movie 4-6, non-glycosylated at Ser31 of TβR Ⅱ.
